# Exploring the key structural attributes and chemico-biological interactions of pyridinone-based SARS-CoV-2 3CL^pro^ inhibitors through validated structure-based drug design strategies

**DOI:** 10.1016/j.heliyon.2024.e40404

**Published:** 2024-11-15

**Authors:** Suvankar Banerjee, Sandip Kumar Baidya, Balaram Ghosh, Tarun Jha, Nilanjan Adhikari

**Affiliations:** a Natural Science Laboratory, Division of Medicinal and Pharmaceutical Chemistry, Department of Pharmaceutical Technology, Jadavpur University, Kolkata, 700032, India; b Epigenetic Research Laboratory, Department of Pharmacy, Birla Institute of Technology and Science-Pilani, Hyderabad Campus, Shamirpet, Hyderabad, 500078, India

**Keywords:** SARS-CoV-2 3CL^pro^ inhibitors, Structure-based molecular modelling, Molecular docking-based QSAR, Field-based 3D-QSAR, Pharmacophore mapping, Drug repurposing

## Abstract

The global outbreak of COVID-19 infection is the first pandemic the world has experienced in this 21^st^ century. The novel coronavirus 2019 (nCoV-19) also called the SARS-CoV-2 is the reason behind the severe acute respiratory syndrome (SARS) that led to this worldwide crisis. In this current post-pandemic situation, despite having effective vaccines, the paucity of orally administrable drug molecules for such infections is a major drawback in this current scenario. Among the different viral enzymes, the SARS-CoV-2 3CL^pro^ is an encouraging target for effective drug discovery and development. In this context, the understanding of the requirements of the small molecules at the active site and their interactions is a crucial aspect of such drug candidate development. Here in this study, structure-based pharmacophore model development and molecular docking-dependent 2D-interaction-based and 3D-field-based QSAR studies have been carried out for a set of potential SARS-CoV-2 3CL^pro^ inhibitors. This study exposed the importance of interactions with amino acids of the active site (such as Leu167 and Gln189 amino acid residues) as well as the importance of hydrogen bond acceptor groups at the S2 and S1′ pockets. The presence of hydrophobic aromatic features as well as hydrophobic contacts at the S1 and S4 pockets were also found to have a key contribution to the SARS-CoV-2 3CL^pro^ inhibition. Moreover, the screened drug candidate Elobixibat from the structure-based virtual screening also explored promising results as evidenced in MD simulation study and thus, can be a promising drug candidate that can be repurposed to assist in the development of effective anti-SARS-CoV-2 therapy.

## 1 Introduction

The first pandemic of the 21^st^ century hit human civilization in 2020, in the form of SARS-CoV-2 infection that turned out into a global disaster in the name of COVID-19, impacting the socio-economic conditions globally [[1], [2], [3]]. For the last two years, severe acute respiratory syndrome (SARS) infection which is an extremely transmissible respiratory tract infection [4] has infected more than 654 million people (654,425,804) to date and has become the reason for the death of over 6.6 million lives globally in around 200 countries [5]. Although the critical period of the COVID-19 pandemic has slowly faded away, the COVID-19 infections and related deaths are still on the loose.

The novel coronavirus 2019 (nCoV-19) also known as the SARS-CoV-2 virus is the main culprit behind this global COVID-19 pandemic. The mapping of the genomic sequence of SARS-CoV-2 identified the virus as a member of the betacoronavirus family those generally known to infect bats [6]. This virus is a positive single-stranded RNA virus that has almost 89% sequence similitude with the SARS-CoV [6,7]. The SARS-CoV-2 virus is the successor of the SARS-CoV as well as the Middle East respiratory syndrome coronavirus (MERS-CoV) that took place between 2000 and 2010. The genomic sequence of SARS-CoV-2 comprises two overlapped open reading frames (ORFs) [[6], [7], [8], [9], [10]]. The translation of these ORFs synthesizes two polypeptide (pp) chains such as pp1a and pp1ab containing 16 non-structural proteins (NSPs) that are crucial for the lifecycle of the SARS-CoV-2 [6,8,9]. In the process of anti-SARS-CoV-2 therapeutic developments, several of the viral proteins such as the 3-chymotrypsin-like protease (3CL^pro^), papain-like protease (PL^pro^), as well as RNA-dependant RNA polymerase (RdRp) along with the angiotensin-converting enzyme II (ACE-II) of the human biology that assists the viral entry to the host cell [11]. The 3CL^pro^ and the PL^pro^ enzymes of the SARS-CoV-2 are two important proteases that cleave the pp chains to generate the NSPs (nsp1-nsp16) [7]. These NSPs are further utilized to complete the life cycle of the virus. Therefore, the 3CL^pro^-mediated proteolytic activity of the pp in NSPs has identified the enzyme as a promising target for anti-SARS-CoV-2 therapeutic developments.

**Fig. 1:**
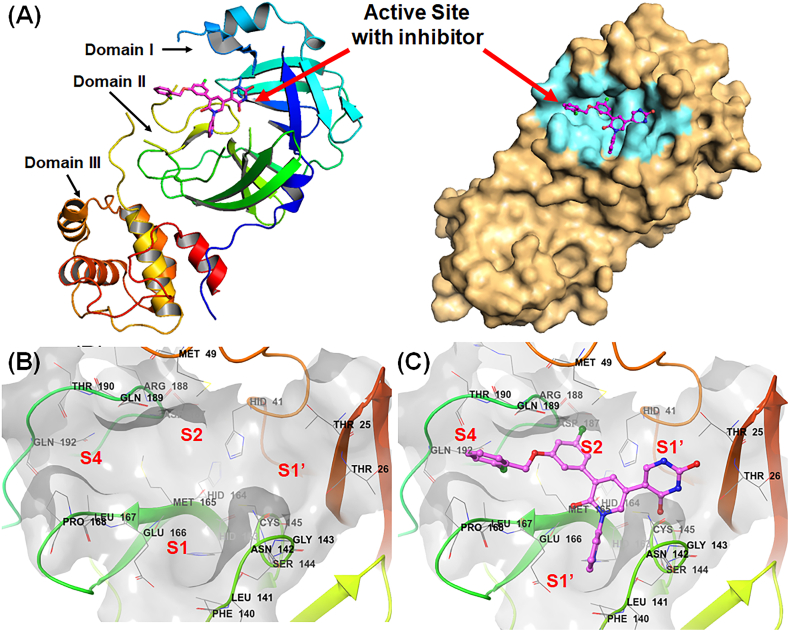
(A) The ribbon and surface representation of the SARS-CoV-2 3CL^pro^ (PDB ID: 7L13) with the inhibitor-bound active site; **(B)** the active site of SARS-CoV-2 3CL^pro^ (PDB ID: 7L13) along with sub-sites; **(C)** non-covalent inhibitor-bound SARS-CoV-2 3CL^pro^ (PDB ID: 7L13) active site with important sub-sites.

The SARS-CoV-2 3CL^pro^ is a 33 kD protein with 306 amino acids that are distributed in three different domains such as domain I (comprising 8–100 amino acids), domain II (101–183 amino acids) and domain III (200–303 amino acids) along with an N-finger motif (1–7 amino acids) to assist the dimerization [8]. Domain I and II together are recognized as the N-terminal domain consisting of 13 β-strands and one anti-parallel β-sheet. Apart from dimerization, the N-finger motif also takes part a crucial role in active site formation [8]. The active site of the SARS-CoV-2 3CL^pro^ is positioned between domain I and domain II of the monomer (Fig. 1A).

**Fig. 2:**
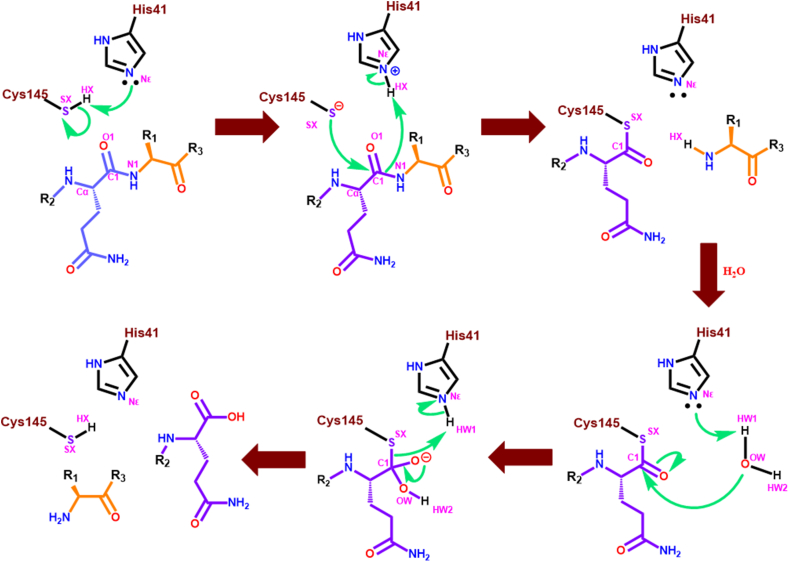
The catalytic mechanism of His41-Cys145 dyad for SARS-CoV-2 3CL^pro^ proteolytic activity.

The active site contains two essential amino acids (His41 and Cys145) that execute the catalytic activity of the enzyme and are known as the catalytic dyad. The catalytic activity of this enzyme is similar to the activity of the Ser-His-Asp catalytic triad of the serine protease family. Upon substrate binding at the active site, His41 prepares the Cys145 for a nucleophilic attack on the substrate by deprotonating the thiol (-SH) function of the Cys145 residue. Due to such deprotonation, Cys145 launches a nucleophilic attack on the glutamine backbone carbon atom of the pp (Fig. 2). This leads to form a tetrahedral thiohemiketal intermediate and subsequently forming an oxyanion. This oxyanion is stabilized by His41 and the nearby Gly143 and Ser144 amide groups. As a result, the thiohemiketal group transforms into a thioester where the peptide bond is broken, and the C-terminal portion of the pp is released [6,12,13]. Finally, a water molecule-mediated hydrolysis cleaves the thioester linkage to liberate the N-terminal part of the pp substrate (Fig. 2). Apart from that, for the development of potent inhibitors, studies related to the SARS-CoV-2 3CL^pro^ elucidated different subsites (S1, S1′, S2, S4 etc.) at the active site with several amino acid residues (Leu167, His164, Glu166, Gln189, Thr190, Gln192) those are crucial for inhibitor binding (Fig. 1**B** and **C**) [6,12,13].

The identification of the structural attributes of small molecule inhibitors is a crucial aspect of the development of effective drug candidates for a specific target. The quantitative structure-activity relationship (QSAR) is referred to as a collection of *in silico* computational methods that utilize different statistical methods. This method correlates the molecular properties of compounds with their biological potential via mathematical means to deduce the salient structural aspects that regulate their biological responses.

Additionally, apart from the well-known covalent inhibitors of the SARS-CoV-2 3CL^pro^ enzyme such as GC-376 that covalently binds with the Cys145 thiol function [14] to disrupt the catalytic function of His41 and Cys145 dyad, the study of non-covalent inhibitors is another crucial field for the generation of oral anti-SARS-CoV-2 agents. Previous studies on SARS-CoV and SARS-CoV-2 derivatives [15] suggested that besides the covalent inhibitors, the non-covalent and/or designed semi-synthetic natural product derivatives can be used for the development of effective anti-SARS-CoV-2 agents. Furthermore, the peptidomimetic and non-covalent inhibitors can provide improved drug-likeliness by optimizing their binding at the active site or via drug repurposing.

Therefore, in this study, a series of non-covalent inhibitors of SARS-CoV-2 3CL^pro^ was subjected to an active site-guided structure and molecular docking-based QSAR study to identify their different structural aspects and requirements for effective inhibition at the active site. Moreover, it was detected that several of the marketed drug molecules aided the therapeutic management of SARS-CoV-2 infection. Drug candidates such as remdesivir, favipiravir, ivermectin, and doxycycline are effectively used for various other diseases conditions and facilitated the treatment of SARS-CoV-2 infected patients in those critical pandemic situations [[16], [17], [18], [19], [20], [21]]. Therefore, in this study, the developed structure-based QSAR analysis and virtual screening of the DrugBank database [22] have been also performed to identify some potential drug molecules that can be further explored as effective SARS-CoV-2 3CL^pro^ inhibitors.

## 2 Materials and methods

### 2.1 Dataset

A set of 38 pyridinone scaffold-containing molecules with a span of *in vitro* SARS-CoV-2 3CL^pro^ inhibitory potential (Supplementary Table 1) were obtained from Jorgensen and co-workers [[23], [24], [25]] to perform the structure-based molecular modelling study and screening.

### 2.2 Dataset preparation

For the preparation of the chemical dataset in the current study, the 2D structures of these pyridinone analogs were sketched with the help of ChemDraw 5.0 software [26]. The Chem 3D pro software [26] was used to transform the 2D structural data of these compounds into their 3D forms. Next, the ‘*prepared ligands for QSAR*’ module of Discovery Studio 3.0. software [27] was utilized for the geometry optimization of these chemical entities. Not only that but before conducting the molecular modelling studies, the SARS-CoV-2 3CL^pro^ inhibitory activities (IC_50_ in nM) of these molecules were converted into respective negative logarithmic values.

### 2.3 Dataset division

Separation of the dataset into the training and test sets is one of the major steps of the QSAR study to evaluate the reliability, robustness, as well as internal and external predictive capability of the QSAR models. Here, in this study, to generate the balanced training and test sets, 25% of the dataset molecules were picked out as the test set molecules by the *Y-based ranking method* [28] while the remaining 75% of compounds comprised both the most active (compound **19**) and the least active (compound **1**) molecules were considered as the training set.

### 2.4 Procurement of the crystal-bound conformers

The protein-bound 3D conformer of a bioactive compound/drug molecule, known as the bioactive conformer, conveys several crucial information regarding the proper binding, and geometrical 3D orientation of that molecule that can assist in drug development and associated studies. Here, from the 38 SARS-CoV-2 3CL^pro^ inhibitors, 13 molecules including the most active compound were found to have 3D SARS-CoV-2 3CL^pro^ enzyme-bound X-ray crystallographic data at the enzyme active site. The information regarding this crystallographic data of the SARS-CoV-2 3CL^pro^-bound pyridinone analogs including their reported SARS-CoV-2 3CL^pro^ inhibitory activities is tabulated in Table 1.

**Table 1:** SARS-CoV-2 3CL^pro^ X-Ray co-crystal-bound dataset compounds [[21], [22], [23]].

PDB ID	Resolution (Å)	Inbound dataset compound no.	SARS-CoV-2 3CL^pro^ IC_50_ (μM)
7L10	1.63	Compound 3	4.020
7L11	1.80	Compound 4	0.140
7M8X	1.74	Compound 5	0.470
7M8M	1.78	Compound 9	0.120
7L12	1.80	Compound 12	0.128
7M8Y	1.75	Compound 13	0.110
7M8N	1.96	Compound 14	0.100
7M8O	2.44	Compound 17	0.037
7L13	2.17	Compound 19	0.018
7M8P	2.23	Compound 21	0.020
7M91	1.95	Compound 23	0.025
7L14	1.80	Compound 24	0.170
7N44	1.94	Compound 31	0.042

### 2.5 Molecular docking study

Apart from the crystal-bound most active molecule for a structure-based study, the rest of the dataset compounds were subjected to a molecular docking analysis at the SARS-CoV-2 3CL^pro^ enzyme active site to acquire knowledge about their bioactive or near bioactive conformations. Here, in this study, the X-ray crystallographic data of the most active compound **19** (**PDB ID**: 7L13) [[23], [24], [25], [26], [27], [28], [29]] was considered for the molecular docking analysis of these compounds utilizing by the Maestro v12.1 software of the Schrodinger suite [30].

#### 2.5.1 Ligand preparation

In this study, the *Ligprep* module incorporated in the Schrodinger Maestro v12.1 [30] was applied for the energy minimization of these dataset compounds by the optimized potential for liquid simulations 2005 (*OPLS2005*) force field [31].

#### 2.5.2 Protein preparation

Primarily, the 3D crystallographic structure of compound **19**-bound SARS-CoV-2 3CL^pro^ (**PDB ID**: 7L13) [29] was utilized. Then the refinement and optimization of the enzyme structure along with hydrogens addition, water molecules removal, assignment of bond order, as well as addition of side chain missing atoms were carried out [32] with the assistance of the ‘*Protein preparation*’ module incorporated in Schrodinger Maestro v12.1 software [30].

#### 2.5.3 Generation of receptor grid

The ‘*receptor grid generation*’ module from the Schrodinger Maestro v12.1 software [30] was utilized to create a rigid grid around the active site of the SARS-CoV-2 3CL^pro^ enzyme. The default grid box of 20 Å was utilized to produce the receptor grid including the inbound ligand kept at the centroid.

#### 2.5.4 Ligand docking

**Fig. 3:**
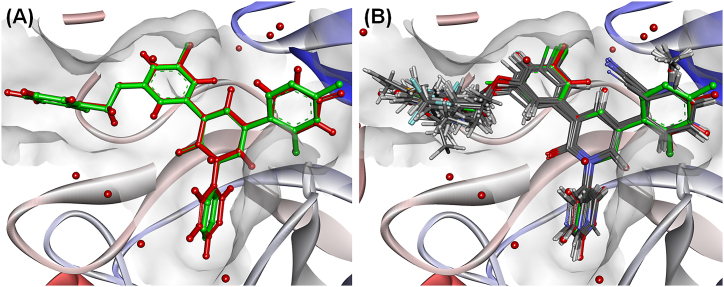
(A) Alignment of the co-crystallized compound **19** (red ball and stick) and its re-docking conformation (green ball and stick) at SARS-CoV-2 3CL^pro^ (PDB ID: 7L13) active site **(B)** Alignment of the inbound compound **19** (red ball and stick) and its re-docked conformation (green ball and stick) along and the other dataset compounds (grey ball and sticks) at SARS-CoV-2 3CL^pro^ (PDB ID: 7L13) active site.

These compounds were predicted by using an ‘*extra precision* (*XP*)’ method with *OPLS2005* forcefield [31], flexible ligand sampling utilizing grid-based Ligand Docking and Sampling (*GLIDE*) module with the help of the *Ligand Docking* protocol of Schrodinger Maestro v12.1 [30 32]. In addition, the interaction scores of these compounds per residue of the active site were computed within a 12 Å radius from the grid centre for further assessment [30,32]. The re-docked inbound molecule (Compound **19**) and the docking conformation of the dataset molecules are given in Fig. 3**A** and **B**, respectively.

### 2.6 Molecular docking interaction-based 2D-QSAR study

In this process, the molecular docking interaction scores, the distance between atoms of the docked compounds and the active site amino acids along with other *GLIDE* [30] docking-generated molecular descriptors were utilized to correlate the SARS-CoV-2 3CL^pro^ inhibitory activity of these molecules [33].

#### 2.6.1 Dataset pre-treatment and model development

For such a study, the molecular docking-based descriptors were procured and subsequently subjected to a dataset pre-treatment process. Here, a correlation cut-off of 0.95 as well as a co-variance threshold value of 0.001 was used to eliminate the highly correlated features [34]. In the step of feature selection, two different methods, i.e., stepwise multiple linear regression (SW-MLR) and the genetic algorithm (GA)-based best subset selection (BSS) methods were adopted [28,34,35]. Finally, the feature sets were selected from the S-MLR, and the GA-based methods, followed by the BSS process [28,34]. The final selected features of the MLR models (SW-MLR and GA-MLR) were developed on the training set compounds. These SW-MLR and GA-MLR models were both tested for their predictive performance by the *LOO-CV*-based internal cross-validation and test set-based external cross-validation [28,35].

#### 2.6.2 MLR model evaluation

Besides calculating the *R*^*2*^, *Q*^*2*^ and the *R*^*2*^_*Pred*_ values, several other internal and external validation parameters such as adjusted *R*^*2*^ (*R*^*2*^_*A*_), standard error of estimate (*SEE*), predicted residual sum of squares (*PRESS*), the Fischer coefficient (*F*) with degree of freedom values were determined to judge the reliability of the MLR models using the DTC lab tools [34]. The robustness of these 2D-QSAR models was tested by the *Y*-scrambling test (50 runs) and *cR*_*p*_^*2*^ calculation [28]. Furthermore, the model acceptability criteria as per Golbraikh and Tropsha [36] and the *r*_*m*_^*2*^ metrics [37] were also ascertained for both of these MLR models developed. Additionally, for both the internal and external validation, other crucial parameters such as the average and delta *r*_*m*_^*2*^ values for the *LOO-CV* and external cross-validation were also calculated. Also, for the external validation, the mean absolute error (*MAE*) of 95% data, the standard deviation of absolute error as well as the predicted root mean square error (*RMSEp*) was calculated for the reported MLR models [34].

### 2.7 Molecular docking-based 3D-QSAR CoMFA and CoMSIA study

The 3D molecular field-based QSAR studies involve the quantification and correlation between the molecular fields (such as steric, hydrophobic, electrostatic fields, etc.) of a group of molecules correlating with their biological activity by aligning or superimposing on a common sub-structure [28,33,[38], [39], [40], [41], [42]].

The comparative molecular field analysis (CoMFA) as well as comparative molecular similarity indices analysis (CoMSIA) are widely acceptable common field-based 3D-QSAR techniques that quantify the molecular fields such as steric, electrostatic, hydrophobic, hydrogen bond donor, and hydrogen bond acceptor fields and finally help to correlate them with the biological potential of the molecules through partial least square (*PLS*) method [28,33,38,39].

From the molecular docking study, the binding poses of these dataset molecules and the X-ray co-crystal-bound the most active compound, i.e., compound **19** (**PDB ID:** 7L13) were considered for performing the 3D-QSAR CoMFA and CoMSIA studies.

For the inspection of the effects and the presence of various molecular fields, the SYBYL-X 2.0 software [43] was used to calculate the steric (*S*) and electrostatic (*E*) CoMFA fields. On the other hand, the steric (*S*), electrostatic (*E*), hydrophobic (*H*), hydrogen bond donor (*HBD*), and hydrogen bond acceptor (*HBA*) fields were computed for the CoMSIA study. The SYBYL-X 2.0 software [43] was used to construct the PLS-based CoMFA and CoMSIA models on the training set compounds and were validated internally with the help of calculating several parameters such as Leave-One-Out (*LOO*) cross-validated *R*^*2*^ (*Q*^*2*^), 10-fold cross-validated *R*^*2*^ (*R*^*2*^_*10-cv*_), bootstrap cross-validated *R*^*2*^ for 20 runs (*R*^*2*^_*20-BS*_) along with further external cross-validation (*R*^*2*^_*Pred*_) for the test set compounds. Furthermore, a *Y*-scrambling test for both the CoMFA and CoMSIA models was executed to examine the reliability and robustness of both these models [28].

### 2.8 Development of common feature pharmacophore hypotheses

Due to the shape and size of the bioactive conformers for the potency, 13 co-crystal-bound pyridinone-based SARS-CoV-2 3CL^pro^ inhibitors (Table 1) were considered as the training set compounds for the construction of structure-based common feature pharmacophore models. For this study, all of these co-crystal-bound training set molecules, and all of these bioactive conformers were aggregated into a single 3D space by superimposing in DS 3.0 software [27]. The available X ray-crystal structures of these SARS-CoV-2 3CL^pro^ inhibitors were retained without any alteration in their specific conformations. The *HipHop* method was used to generate the pharmacophore hypotheses [[44], [45], [46]]. Here, five different pharmacophore features namely hydrogen bond donor (*D*), hydrogen bond acceptor (*A*), ring aromatic (*R*), hydrophobic (*H*) and hydrophobic aromatic (*Z*) were considered for building the common feature pharmacophore hypotheses. The ‘*principal*’ and ‘*MaxOmitFeat*’ values of 0, 1 and 2 were set for the training set compounds with no conformation generation to allow partial and no mapping of the bioactive conformers of the training set compounds [27].

Additionally, the validation of the constructed hypothesis was performed using 25 remaining SARS-CoV-2 3CL^pro^ inhibitors (considered as *Actives*) and a decoy dataset of 1231 decoy molecules (considered as *Inactives*) obtained from the DUD-E database [47,48]. A ‘*BEST*’ conformation generation with the ‘*Flexible*’ fitting method was used while retaining a default maximum conformations generation value of 250 and conformation generation energy threshold of 20 kcal/mol along with a ‘*Maximum Omitted feature’* value of 0 [27].

For these developed pharmacophore hypotheses, the goodness-of-hit (GH), Ranking, %Y, and %A scores along with their sensitivity and specificity values were considered for the best hypothesis selection [46].

### 2.9 Structure-based virtual screening (SBVS) of drug and drug-like molecules

In this scarcity of budding drug candidates for effective anti-SARS-CoV-2 therapy, the repurposing of available drug molecules such as remdesivir, doxycycline and favipiravir as well as ivermectin-like drugs provided a reinforced treatment for SARS-CoV-2 infection in the pandemic situation. Therefore, the identification of potential SARS-CoV-2 inhibitors from available drugs within a short period and drug-like lead molecules is an achievable target for such therapeutic advancements.

Hence, in this study, the developed structure-based studies conducted on these non-covalent SARS-CoV-2 3CL^pro^ inhibitors were considered for the screening of potential lead drug and drug-like molecules from the DrugBank database [22]. These drugs and drug-like molecules were initially screened depending on the constructed pharmacophore hypothesis (***Hypo-1***) followed by a molecular docking study at the SARS-CoV-2 3CL^pro^ enzyme active site. Finally, the molecular docking interaction-dependent QSAR models were used to predict the probable inhibitory potential. This technique may assist in the identification of the potential final lead drug/drug-like molecules for SARS-CoV-2 3CL^pro^ inhibitor development.

### 2.10 Molecular dynamics (MD) simulation study

To further validate the binding stability of the screened lead molecules including the SARS-CoV-2 3CL^pro^ (**PDB ID**: 7L13) and the co-crystallized pyridinone derivative (compound **19)** were performed with the help of *DESMOND* module available in Schrodinger Maestro v12.1 [30] software for 100ns timeframe.

#### 2.10.1 Protein-ligand complex preparation

The *Protein preparation*’ module of Schrodinger Maestro v12.1 software [30] was utilized to prepare the SARS-COV-2 3CL^pro^ (**PDB ID:** 7L13)-compound **19** (co-crystallized ligand) and SARS-CoV-2 3CL^pro^ (**PDB ID:** 7L13)-screened compound complexes. The removal of co-crystallized water molecules, protonation, bond order assignment, as well as the addition of missing amino acids and side chain residues were performed for each of the complexes at a pH of 7.0 (±2.0). Also, the *PROPKA* module and the *OPLS2005* forcefield [31] were utilized to optimize and the minimization of the protein-ligand (P-L)-complexes similar to the molecular docking study conducted earlier.

#### 2.10.2 System grid generation

To generate cubic systems for the P-L complexes with *TIP3P* water model and a buffer distance to the periodic boundary of 10 Å were built in the *“System Builder”* application. The system was neutralized via introducing the required amount of Na^+^/Cl^−^ ions. Additionally, an isotonic condition (NaCl conc. of 0.15 M) was maintained through the incorporation of Na^+^ and Cl^−^ ions while excluding salt and ion placement within 20 Å of the bound ligands whereas the systems were generated by utilizing the *OPLS2005* forcefield.

#### 2.10.3 Molecular dynamics (MD) simulation

Each of the 100ns MD simulation studies was carried out with the help of the *DESMOND* module of the Schrodinger Maestro v12.1 software [30]. Prior to the MD simulation study, a system relaxation protocol was conducted using *RESPA* integrator with a 2fs timestep. Additionally, the *Nose-Hoover chain* thermostat at 310.15K temperature with 1fs timestep and isotropic coupling-based *Martyna-Tobias-Klein* barostat at 1atm (1.0.13245 bar) pressure with 2fs timestep were selected for the relaxation protocol. Finally, each of the simulations was carried out using the *OPLS2005* forcefield and *NVT* ensembling method [32].

## 3 Results and discussion

It is quite interesting to note that, researchers had reported 13 of these pyridinone-based SARS-CoV-2 3CL^pro^ inhibitors that provide more accurate bioactive conformation of these congeneric series molecules than *in silico* bioactive conformation prediction including the most effective compound (compound **19**) of the series (**PDB ID:** 7L13). Therefore, the rest of the dataset molecules were subjected to a molecular docking study to identify the possible bioactive conformers that provided an excellent alignment of the pyridinone core. Therefore, the interactions and distances of such bioactive conformers from the catalytic site residues can be used to correlate their interaction energies with their SARS-CoV-2 3CL^pro^ inhibitory potency via QSAR and molecular modelling studies to understand the key structural attributes of the P-L complexes that influence their SARS-CoV-2 3CL^pro^ binding and inhibitory activity.

### 3.1 Molecular docking-based QSAR study

The molecular docking interaction-derived descriptors along with the docking interaction energies as well as docking scores were utilized for the construction of QSAR models. The values of the descriptors for these final MLR models (Equation 1, Equation 2) are given in Supplementary Table 2. Statistically validated stepwise MLR (SW-MLR) and genetic algorithm MLR (GA-MLR) models were generated (Equation 1, Equation 2, respectively).

Equation 1 $$\mathrm{pI}C_{50}=1.8388\;\left(\pm 1.462\right)\;‐\;0.90817\;\left(\pm 0.27551\right)\;r\_\mathrm{glide}\_\mathrm{res}:A41\_\mathrm{vdw}\;‐\;0.30911\;\left(\pm 0.07686\right)\;r\_\mathrm{glide}\_\mathrm{res}:A167\_\mathrm{dist}\;‐\;0.37003\;\left(\pm 0.0635\right)\;r\_\mathrm{glide}\_\mathrm{res}:A189\_\mathrm{vdw}$$

Equation 2 $$\mathrm{pI}C_{50}=‐\;0.79824\;\left(\pm 1.79427\right)\;‐\;0.56307\;\left(\pm 0.25248\right)\;r\_\mathrm{glide}\_\mathrm{res}:A41\_\mathrm{vdw}\;‐\;0.09204\;\left(\pm 0.01858\right)\;r\_i\_\mathrm{glide}\_\mathrm{evdw}\;‐\;0.25357\;\left(\pm 0.11531\right)\;r\_\mathrm{glide}\_\mathrm{res}:A192\_\mathrm{dist}$$

The SW-MLR (Equation (1)) model showed the importance of descriptors like *r_glide_res:A41_vdw*, *r_glide_res:A189_vdw* and *r_glide_res:A167_dist* whereas the GA-MLR model (*Equation (*2*)*) exhibited negative impact of descriptors like *r_glide_res:A41_vdw*, *r_i_glide_evdw* and *r_glide_res:A192_dist*. All these descriptors exhibited a negative impact on SARS-CoV-2 3CL^pro^ inhibitory activity. It was fascinating to perceive that both these models exhibited more or less comparable results as far as their statistically validated internal and external validation parameters were concerned (Table 2). Furthermore, the acceptability of these constructed final models was also evaluated by calculating the Golbraikh and Tropsha model acceptability criteria (Table 3) [36].

**Table 2:** Statistical validation parameters for molecular docking-based MLR models.

Parameter	SW-MLR (equation (1))	GA-MLR (equation (2))
*N*_*Train*_	29	29
*SEE*	0.352	0.323
*R*^*2*^	0.753	0.792
*R*^*2*^_*A*_	0.723	0.767
*PRESS*	3.104	2.613
*F (3, 25)*	25.340	31.663
*p-value*	<0.05	<0.05
*Q*^*2*^	0.685	0.696
*Avg. rm*^*2*^_*LOO*_	0.566	0.589
_*C*_*R*_*p*_^*2*^	0.708	0.737
*Avg. Q*^*2*^_*Y-Scramble*_	−0.239 (±0.227)	−0.209 (±0.216)
*N*_*Test*_	09	09
*RMSE*_*P*_	0.375	0.387
*R*^*2*^_*pred*_*(Q*^*2*^*f*_*1*_*)*	0.629	0.605
*Q*^*2*^*f*_*2*_	0.626	0.601
*Avg. rm*^*2*^_*Test*_	0.501	0.512

**Table 3:** Golbraikh and Tropsha model acceptability criteria of the MLR models.

Parameters	Threshold	SW-MLR	GA-MLR
*Q*^*2*^	*Q*^*2*^ > 0.5	0.685	0.696
*r*^*2*^	*r*^*2*^ > 0.6	0.633	0.632
*|r0*^*2*^ – *r’0*^*2*^*|*	*|r0*^*2*^ – *r’0*^*2*^*|* < 0.3	0.156	0.025
*k*	0.85 < *k* < 1.13	0.994	0.998
*(r*^*2*^ - *r0*^*2*^*)/r*^*2*^	*(r*^*2*^ - *r0*^*2*^*)/r*^*2*^ < 0.1	0.003	0.048
*k’*	0.85 < *k’* < 1.15	1.004	0.998
*(r*^*2*^ – *r’0*^*2*^*)/r*^*2*^	*(r*^*2*^ – *r’0*^*2*^*)/r*^*2*^ < 0.1	0.249	0.087

**Fig. 4:**
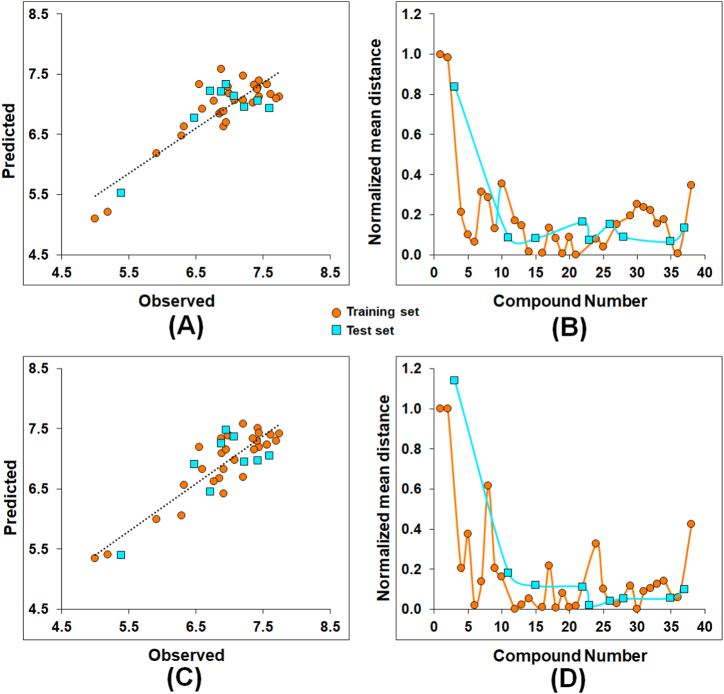
(A) Observed versus predicted activity for the SW-MLR (Equation (1)) model; **(B)** Euclidean-distance-based normalized mean distance values for the SW-MLR (Equation (1)) model; **(C)** Observed versus predicted activity for the GA-MLR (Equation (2)) model; **(D)** Euclidean-distance-based normalized mean distance values for the GA-MLR (Equation (2)) model.

The observed vs. predicted activities for both the SW-MLR and GA-MLR models (Fig. 4**A** and **C**, respectively) and their Euclidean distance-dependent normalized mean distance values for the dataset molecules are depicted in Fig. 4**B** and **D**, respectively. Moreover, the observed activity and respective predicted activity values of the compounds by SW-MLR and GA-MLR models are listed in Supplementary Table 3.

**Fig. 5:**
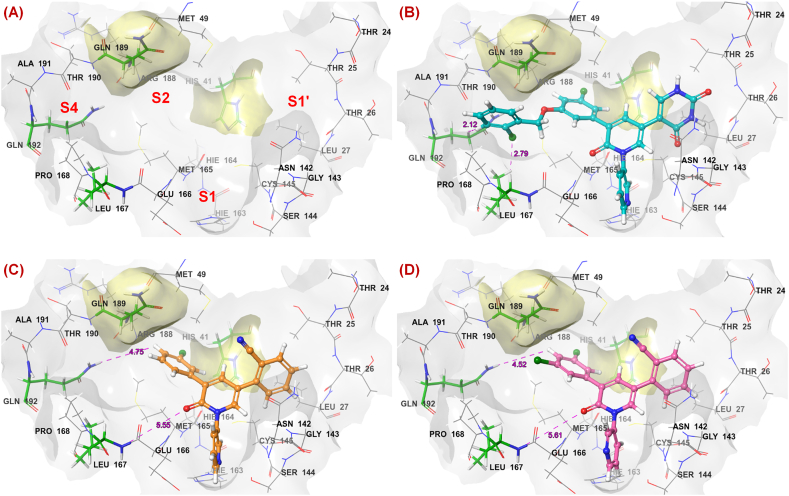
(A) Amino acids (green sticks and yellow surfaces) identified as important for interaction at the active site; **(B)** The distances between the crucial amino acids and the most active compound **19** (cyan ball and stick); **(C)** The distances between the crucial amino acids and the least active compound **1** (orange ball and stick); **(D)** The distances between the crucial amino acids and the lower active compound **3** (pink ball and stick).

It is important to note that compounds **1**–**3** possess almost the lower SARS-CoV-2 3CL^pro^ inhibitory efficacy. Interestingly, compound **1** is the lowest active molecule. The SAR analysis revealed that the activity increased when bulky longer substitutions were done on the R position. However, compounds **1**–**2** did not possess any substitution, and compound **3** possessed only a chlorine substitution at this position. The molecular docking study (Fig. 5**B**–**D**) also revealed that the bulky elongated substitution (such as benzyloxy for compound **19**) is needed for interacting with amino acid residues at the S4 pocket which is not present for compounds without any substitution (compound **1**) and smaller substituents like chloro at this position (compound **3**). Thus, no substitution at the R position is unfavourable for maintaining higher efficacy. Therefore, these lower effective molecules (compounds **1**–**3**) were found clustered separately in a lower region of these plots (Fig. 4**A** and **C**).

Both of these models displayed the negative impact of the van der Waals interaction of amino acid residue His41 towards SARS-CoV-2 3CL^pro^ inhibition (Fig. 5A).

As the value of the descriptor *r_glide_res: A41_vdw* was negative, it suggested that the lower negative value of the van der Waals interaction imparted by His41 may be beneficial to the biological potency. It was discerned that for the most active compound **19**, the central pyridinone ring along with the pyrimidinedione scaffold directed towards the S1′ pocket (R_3_ position) may exert favourable van der Waals interaction with His41 residue (Fig. 5B). Many of these molecules in the dataset (compounds **16**, **20**, **22**, **23**, **28**, **30**, **31**, **34**, **35** and **38**) exhibited the higher negative values of this descriptor and therefore, may exert the higher SARS-CoV-2 3CL^pro^ inhibitory activity. It was also fascinating to note that molecules bearing pyrimidinedione or substituted pyrimidinedione ring system at the R_3_ position exhibited better efficacy compared to the respective cyanophenyl substitution at the same position. The least active compound **1** bearing a cyanophenyl group at the R_3_ position had the lower negative value of this descriptor. Similarly, other molecules (compounds **2**–**7**) were also found to possess the lower negative value of this descriptor. Probably, due to the presence of a cyanophenyl ring, these molecules moved in the other direction and there was less chance of forming a van der Waals interaction.

Similarly, van der Waals interaction imparted by Gln189 at the S2 subsite may be a crucial factor for the biological activity modulation. Molecules having the higher negative values of the descriptor *r_glide_res: A189_vdw* (compounds **14**–**21**, **27**, **30**–**34**, **36**, and **38**) exhibited a greater SARS-CoV-2 3CL^pro^ inhibition compared to lower negative values of this descriptor (compounds **1**–**5**, **8**, **10**–**11**, and **24**). The phenyl or substituted phenyl ring at the core pyridinone moiety may be responsible for forming van der Waals interaction with Gln189 at the S2 pocket. Compound **19** showed that the *o*-chlorobenzyloxy phenyl group was extended in between the S2 and S4 pockets and may provide proper van der Waals interaction for the higher SARS-CoV-2 inhibition. The distance of the molecule from Leu167 was also a crucial factor in the modulation of the activity. It suggested proper positioning of the compound near the S4 pocket. Interestingly, molecules having a distance range of 2.5 Å-3.5 Å were more potent (compounds **16**–**24**, **36**, and **38**) than molecules containing higher distance values (compounds **1**–**3**, and **8**). A closer distance from Leu167 may indicate some favourable interactions for higher inhibition.

In the case of the GA-MLR model (*Equation 2*), apart from the descriptor *r_glide_res: A41_vdw*, two other important parameters were *r_i_glide_evdw* and *r_glide_res: A192_dist*. Molecules having the higher negative value of *r_i_glide_evdw* were potent SARS-CoV-2 3CL^pro^ inhibitors (compounds **16**–**21**, **31**, **35**–**36**, and **38**). It suggested that these compounds were capable of forming higher van der Waals interactions favourable for the higher inhibitory efficacy. Similarly, molecules with a lower value of this parameter were the lower effective SARS-CoV-2 3CL^pro^ inhibitors (compounds **1**–**10**). Again, molecules having a lower distance from the Gln192 amino acid residue close to the S4 pocket were potent inhibitors (compounds **16**–**23**, **30**, **33**, and **35**). For the least active and lower active compounds **1** and **3**, respectively, it was noticed that the distance between the molecules and the Gln187 and Leu167 residues is higher with respect to the most active compound **19** (Fig. 5). Therefore, it may be assumed that compounds containing elongated side chains directed towards the S4 subsite may execute favourable interactions with Gln192 residue conducive to the activity.

### 3.2 Molecular docking-based 3D-QSAR CoMFA and CoMSIA study

#### 3.2.1 Comparative molecular field analysis (CoMFA)

The molecular docking-based three-dimensional alignments of all these compounds were taken into consideration for CoMFA and CoMSIA analysis. Regarding the CoMFA model, it produced acceptable internal and external predictability (*Q*^*2*^ = 0.703; *R*^*2*^_*10-CV*_ = 0.599; *R*^*2*^_*20-BS*_ = 0.973 and *R*^*2*^_*Pred*_ = 0.791) (Table 4).

**Table 4:** Statistical validation parameters for the CoMFA and CoMSIA models.

Parameters	CoMFA	CoMSIA
*Features*	*S, E*	*S, E, H, A*
*Q*^*2*^	0.703	0.694
*Component*	4	5
*R*^*2*^	0.954	0.929
*SEE*	0.156	0.196
*R*^*2*^_*10-cv*_	0.599	0.694
*R*^*2*^_*20-BS*_	0.973	0.956
*F*	123.276 (4, 24)	60.390 (5, 23)
*R*^*2*^_*5cr*_	0.369	0.329
*CSDEP*	0.551	0.594
dq^2^/dr_yy_^2^	2.534	2.98
*R*^*2*^_*pred*_	0.791	607

***Field***	***Field distribution (%)***	

*Steric*	51.4	17.7
*Electrostatic*	48.6	35.4
*Hydrophobic*	–	30.2
*Hydrogen bond acceptor*	–	16.7

**Fig. 6:**
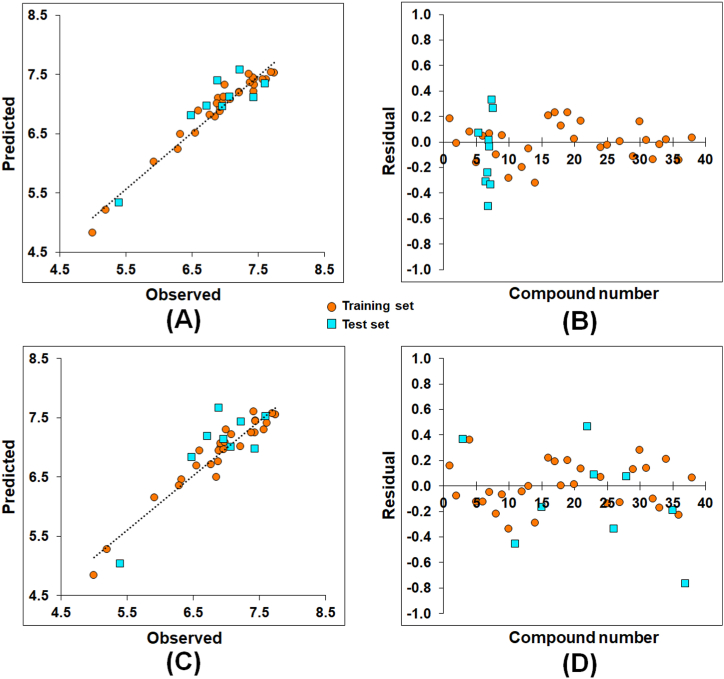
(A) Observed versus predicted activity for the CoMFA model; **(B)** Residual error values for the CoMFA model; **(C)** Observed versus predicted activity for the CoMSIA model; **(D)** Residual error values for the CoMSIA model.

The observed versus predicted values as well as the residual values for the CoMFA model are provided in Fig. 6**A** and **B**, respectively. Also, the observed and CoMFA-predicted activity values are provided in Supplementary Table 3.

**Fig. 7:**
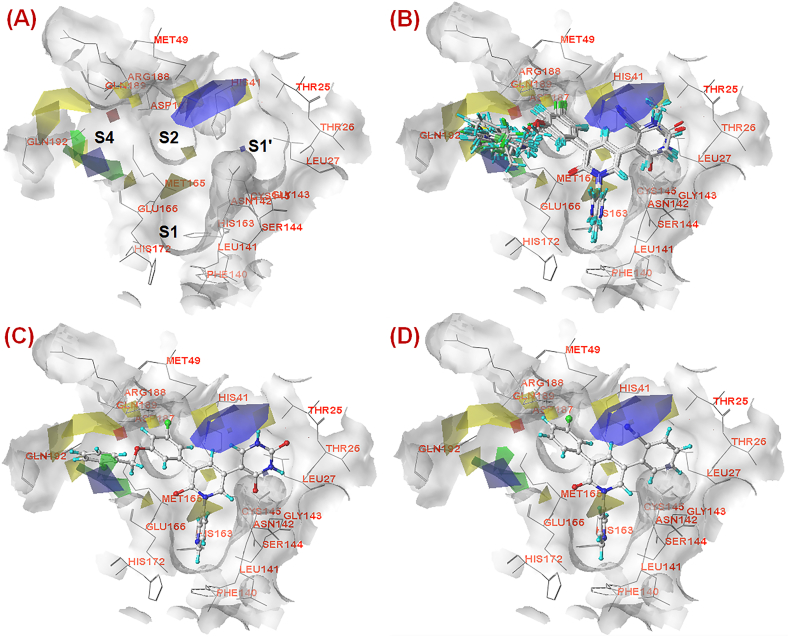
(A) CoMFA contours at the active site of SARS-CoV-2 3CL^pro^ (PDB ID: 7L13); **(B)** CoMFA contours at the active site of SARS-CoV-2 3CL^pro^ (PDB ID: 7L13) with dataset compounds; **(C)** CoMFA contours at the active site of SARS-CoV-2 3CL^pro^ (PDB ID: 7L13) with the most active compound **19**; **(D)** CoMFA contours at the active site of SARS-CoV-2 3CL^pro^ (PDB ID: 7L13) with the least active compound **1**.

The outcomes of the field-based 3D-QSAR studies are in agreement with the outcomes of the MLR models (Equation 1, Equation 2) for the lower active molecules (compounds **1**–**3**). The CoMFA model revealed 51.40% and 48.60% steric and electrostatic field distribution, respectively. The CoMFA contour plot (Fig. 7**A**) revealed some favourable and unfavourable steric and electrostatic fields mainly at the S1′, S2 and S4 pockets.

The contour plot pointed out that a favourable electrostatic contour (blue polyhedron contour) was located near the pyrimidinedione scaffold at the R_3_ position as well as near the central pyridinone scaffold (Fig. 7**B**). It indicated clearly that substitution with groups exerting favourable electrostatic interactions at these positions may be favourable for the greater SARS-CoV-2 3CL^pro^ inhibition. Another favourable electrostatic region (blue contour) was found near the S4 pocket. The most active compound (compound **19**) exhibited that the *o*-chlorobenzyl ring was situated close to the electrostatic favourable region (Fig. 7**C**). It suggested that substitutions exerting electrostatic favourability at the S4 pocket may be crucial for the higher inhibitory potency. On the other hand, a small electrostatic unfavourable region (red contour) found away from the compound suggested that this unfavourable electrostatic contour has no such effect on SARS-CoV-2 3CL^pro^ inhibitory efficacy.

Apart from that, a steric favourable region (green contour) was found near the benzyl group at the S4 pocket. It suggested that smaller substitution with steric effect at the S4 pocket may be conducive to inhibitory efficacy. However, the bigger unfavourable steric region (yellow contour) near the benzyl moiety of compound **19** implied that bulky steric substitution may have detrimental effects on SARS-CoV-2 3CL^pro^ inhibition (Fig. 7**C**). It was also interesting to notice that substitution with unfavourable steric effects at S1′, S1 and S2 regions may be detrimental to SARS-CoV-2 3CL^pro^ inhibition. For the least active molecule (compound **1**), it was noted that the cyano group directly entered into the electrostatic favourable region (blue contour) (Fig. 7**D**). However, the absence of elongated aryl or heteroaryl substitution directed towards the S4 pocket may be the major reason for the lowest efficacy. A similar observation was also noticed for the other lower active molecule (compound **3**).

#### 3.2.2 Comparative molecular similarity indices analysis (CoMSIA)

Regarding the CoMSIA model, it also produced acceptable internal and external predictability (*Q*^*2*^ = 0.694; *R*^*2*^_*10-CV*_ = 0.694; *R*^*2*^_*20-BS*_ = 0.956 and *R*^*2*^_*Pred*_ = 0.607) (Table 4). The observed versus predicted activity values as well as the residual values for the CoMSIA model are provided in Fig. 6**C** and **D**, respectively. Additionally, the observed and CoMSIA-predicted activity values are provided in Supplementary Table 3. The CoMSIA model revealed 17.70%, 35.40%, 30.20%, and 16.70% steric, electrostatic, hydrophobic, and hydrogen bond acceptor field distribution, respectively.

**Fig. 8:**
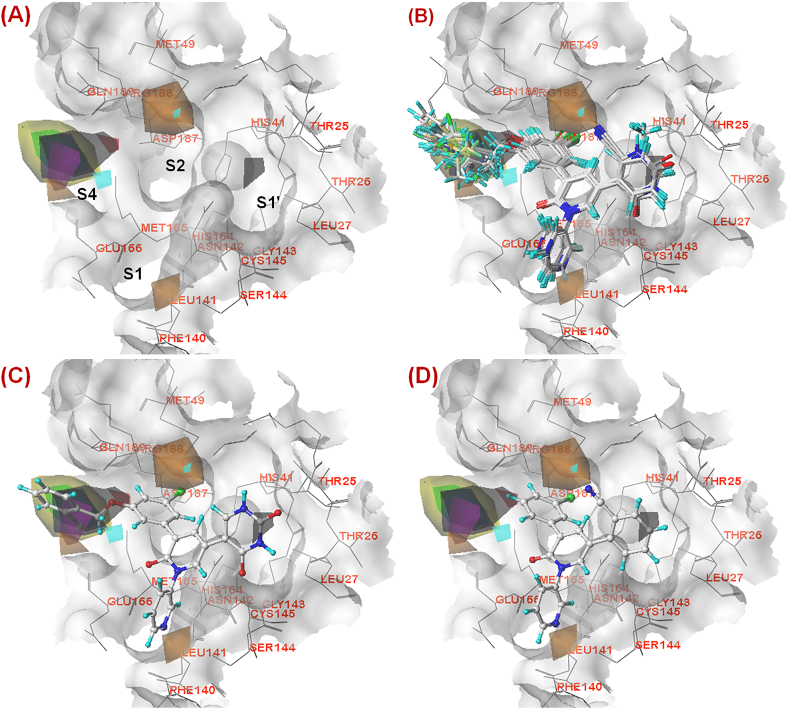
(A) CoMSIA contours at the active site of SARS-CoV-2 3CL^pro^ (PDB ID: 7L13); **(B)** CoMSIA contours at the active site of SARS-CoV-2 3CL^pro^ (PDB ID: 7L13) with dataset compounds; **(C)** CoMSIA contours at the active site of SARS-CoV-2 3CL^pro^ (PDB ID: 7L13) with the most active compound **19**; **(D)** CoMSIA contours at the active site of SARS-CoV-2 3CL^pro^ (PDB ID: 7L13) with the least active compound **1**.

It was interesting to note that the steric features obtained from the CoMSIA study were almost similar to the steric features obtained in the CoMFA study (Fig. 7**A**–**B** and Fig. 8**A**–**B**).

For the contour map of the most active molecule (compound **19**, Fig. 8**C**), it was observed that smaller steric substitution at the benzyl moiety at the S4 pocket may be favourable but bulky steric substitution may have detrimental effects on inhibitory activity. Nevertheless, it was also observed that a smaller hydrophobic substitution (magenta contour) at the S4 pocket may be favourable but a bulky hydrophobic substitution (black contour) may be unfavourable for the activity. Again, hydrophobic substitution at the S1′ pocket may not be favourable for SARS-CoV-2 3CL^pro^ inhibition as seen by the smaller black contour. It was also noted that hydrogen bond acceptor groups near the phenyl ring in between S2 and S4 pockets may favour the activity (cyan contour) whereas such groups were found unfavourable near S1, S2 and S4 pockets as seen by several orange contours at these positions. The CoMSIA analysis (Fig. 8) also reflected that for all these compounds comprising the *o*-cyanophenyl group, the cyano group was located closer to the unfavourable hydrogen bond acceptor region (orange polyhedron) that was not observed for other compounds comprising the pyrimidinedione scaffold. Therefore, it may be suggested that pyrimidinedione scaffold is better than the *o*-cyanophenyl scaffold. Comparing the contour plots of the most active (compound **19**, Fig. 8**C**) and the least active (compound **1**, Fig. 8**D**) molecules, it may be assumed that aryl or heteroaryl substitution with optimum size directed to the S4 pocket may have crucial roles in modulating the SARS-CoV-2 3CL^pro^ inhibitory efficiency.

### 3.3 Common feature pharmacophore mapping

The common feature pharmacophore mapping study resulted in 10 hypotheses comprising 6 pharmacophoric features in each model. Importantly, all these hypotheses passed the acceptable model criteria (GH score >0.6) (Table 5) [45].

**Table 5:** Summary of the common feature pharmacophore models.

Hypo	Se	Sp	D	A	H_a_	H_t_	E	%Y	%A	FP	FN	GH Score	Ranking	Features	MaxFit
1	0.920	0.990	1256	25	23	35	33.015	65.714	92	12	2	0.716	180.237	RZHHAA	6
2	0.960	0.987	1256	25	24	40	30.144	60.000	96	16	1	0.681	180.237	RZHHAA	6
3	0.920	0.989	1256	25	23	37	31.230	62.162	92	14	2	0.688	179.303	RRHHAA	6
4	0.920	0.989	1256	25	23	36	32.098	63.889	92	13	2	0.702	179.303	RRHHAA	6
5	0.920	0.989	1256	25	23	36	32.098	63.889	92	13	2	0.702	178.681	RHHAAA	6
6	0.920	0.994	1256	25	23	31	37.275	74.194	92	8	2	0.781	178.681	RHHAAA	6
7	1.000	0.985	1256	25	25	44	28.545	56.818	100	19	0	0.666	172.659	RRHHAA	6
8	1.000	0.984	1256	25	25	45	27.911	55.556	100	20	0	0.656	172.659	RRHHAA	6
9	0.960	0.985	1256	25	24	43	28.041	55.814	96	19	1	0.648	170.637	RHHHAA	6
10	0.920	0.988	1256	25	23	38	30.408	60.526	92	15	2	0.676	170.637	RHHHAA	6

R = Ring aromatic, Z = Hydrophobic aromatic, H = Hydrophobic, A = H-bond Acceptor.

The best hypothesis (*Hypo-1*) is depicted in italics and bold.

**Fig. 9:**
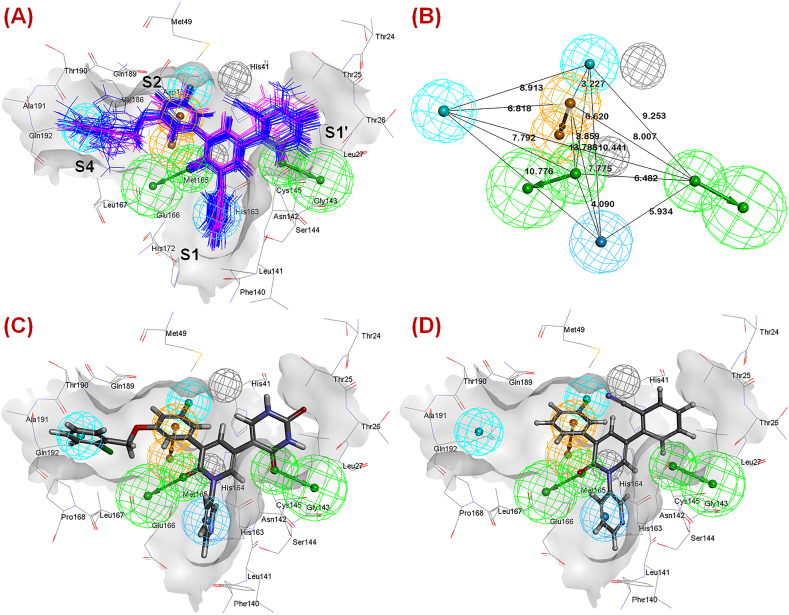
(A) *Hypo-1* model showing mapped dataset compounds (magenta lines: co-crystal-bound dataset compounds; blue lines: docked conformations of rest of the dataset compounds) at the active site of SARS-CoV-2 3CL^pro^ (PDB ID: 7L13); **(B)** Distance between the pharmacophore features according to *Hypo-1* model; **(C)** The most active compound **19** mapped in the *Hypo-1* model; **(D)** The least active compound **1** mapped in the *Hypo-1* model.

However, among these hypotheses, hypothesis 1 (***Hypo-1***) was selected as the best one due to the highest ranking (180.237) compared to other hypotheses. ***Hypo-1*** exhibited the importance of one ring aromatic (*R*), one hydrophobic aromatic (*Z*), two hydrophobic (*H*) and two hydrogen bond acceptor (*A*) features. It was interesting to note that all these compounds properly fit into the pharmacophoric features in an aligned fashion (Fig. 9**A**).

Again, it was noticed that the two hydrophobic (*H*) features and one hydrophobic aromatic (*Z*) feature were located at the S1, S2 and S4 pockets at a distance of 10.776 Å, 10.441 Å and 8.913 Å, respectively (Fig. 9**B**). It was noticed that for all these molecules, the aryl group attached to the central ring of heteroaryl nitrogen atom was perfectly positioned into the hydrophobic (*H*) feature at the S1 pocket. However, the halogen substitution at the S2 pocket properly fitted into the *H* feature. Importantly, it was noticed that compounds having aryl substitution directed towards the S4 pocket were better potent SARS-CoV-2 3CL^pro^ inhibitors. Again, it was observed that the two hydrogen bond acceptor (*A*) features were located within a distance of 6.482 Å (Fig. 9**B**). The most potent molecule (compound **19**, Fig. 9**C**) having the carbonyl function at the extended heteroaryl moiety directed towards the S1′ pocket accommodated properly with the *A* feature. On the other hand, another carbonyl function of the central heteroaryl scaffold fitted nicely at the *A* feature between the junction of the S1 and S4 pockets. For all these compounds, it was noticed that the phenyl ring directed towards the S2 pocket fitted well into the ring aromatic (*R*) feature suggesting the importance of ring aromaticity exerted by the scaffold for the higher inhibitory potency. Generally, this phenyl group fitted into the ring aromatic (*R*) feature connected the two hydrophobic (*H*) features at the S2 and S4 pockets within 3.227 Å and 6.818 Å, respectively. Molecules containing elongated aryl or heteroaryl groups directed toward the S4 pocket (compounds **16**–**21**, **33**, **36**, and **38**) were better potent than molecules containing smaller groups or without such groups (compounds **1**–**11**, Fig. 9**D**). Therefore, the importance of such elongated aryl or heteroaryl groups directed toward the S4 pocket was implicated for higher SARS-CoV-2 3CL^pro^ inhibition.

### 3.4 Structure-based virtual screening (SBVS) of drug and drug-like molecules

For the *VS* of drug molecules, 11,300 DrugBank database molecules [22] were used for screening in search of promising drug candidates as SARS-CoV-2 3CL^pro^ inhibitors. In this *VS* process, the compounds were cleaned primarily and subsequently prepared by utilizing the ‘*Prepare ligands for QSAR*’ module of DS 3.0 software [27] followed by a duplicate compound check that yielded 11,005 compounds. The prepared molecules were primarily screened with the help of the constructed pharmacophore model (***Hypo-1***). The drug molecules having a *Fit* value ≥ 4.0 were considered for the next screening process. The *Ligand Pharmacophore Mapping* module from the DS 3.0 software [27] was used for the study with the *Flexible* fitting method and a *Maximum Omitted Features* value of *-1*. From this process, a total of 72 eligible drug candidates were selected for further study [27].

**Fig. 10:**
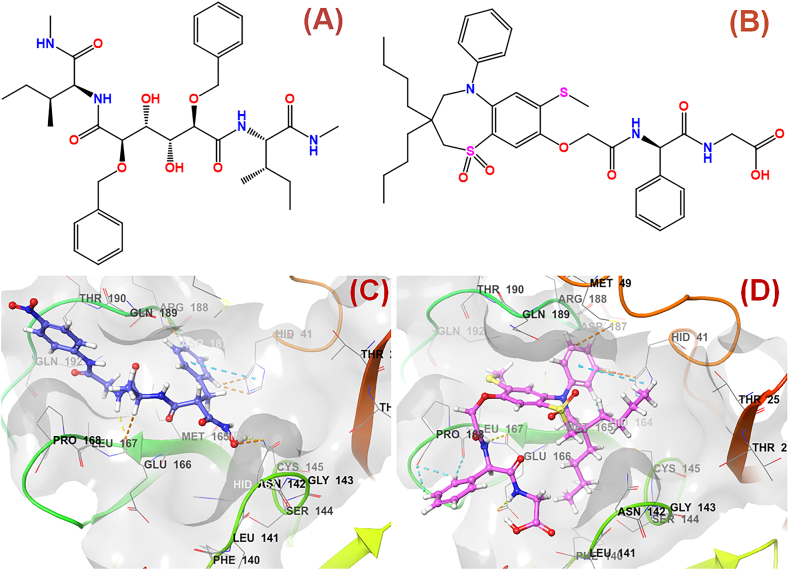
2D Structure of compounds **(A)** N,N-[2,5-O-[Dibenzyl]-glucaryl]-DI-[isoleucyl-amido-methane] (DB03908); **(B)** Elobixibat (DB12486); **(C)** Binding interactions of N,N-[2,5-O-[Dibenzyl]-glucaryl]-DI-[isoleucyl-amido-methane] (DB03908) at the active site of SARS-CoV-2 3CL^pro^ (PDB ID: 7L13).; **(D)** Binding interactions of Elobixibat (DB12486) at the active site of SARS-CoV-2 3CL^pro^ (PDB ID: 7L13).

In the following step, the pharmacophore-screened 72 drug molecules were subjected to a molecular docking study utilizing the *GLIDE* module of Schrodinger Maestro v12.1 [30]. A similar protocol adopted earlier in this study had been applied to these drug molecules. From this step, depending upon their binding pattern and interactions at the SARS-CoV-2 3CL^pro^ (PDB ID: 7L13) active site, 19 drug compounds were selected for further analysis. Next, the molecular docking-based SW-MLR and GA-MLR models were utilized to predict the SARS-CoV-2 3CL^pro^ inhibition data of these 19 screened drug compounds. Finally, the drug candidates showed good SARS-CoV-2 3CL^pro^ inhibitory activity predicted well by both these regression models. Two final molecules, i.e., N,N-[2,5-O-[Dibenzyl]-glucaryl]-DI-[isoleucyl-amido-methane] (DB03908, Fig. 10**A**) and Elobixibat (DB12486, Fig. 10**B**) were selected as the final lead drug molecules that can be repurposed for further development of potential anti-SARS-CoV-2 agents. Elobixibat, marketed as an ileal bile acid transporter inhibitor, is used for the management of chronic constipation by enhancing colonic bile acid concentrations and triggering bile functions. The interactions of DB03908 and DB12486 at the SARS-CoV-2 3CL^pro^ active site amino acid residues are given in Fig. 10**C** and **D**, respectively. The predicted SARS-CoV-2 3CL^pro^ inhibitory activities of these final screened lead molecules are given in Table 6.

**Table 6:** Predicted SARS-CoV-2 3CL^pro^ inhibitory activity of the final screened molecules.

Drug candidate	DrugBank-Database ID	Predicted SARS-CoV-2 3CL^pro^ inhibitory activity (nM)	
		SW-MLR (Equation (1))	GA-MLR (Equation (2))
N,N-[2,5-O-[Dibenzyl]-glucaryl]-DI-[isoleucyl-amido-methane]	DB03908	119.199	7439.78
Elobixibat	DB12486	982.601	979.513

### 3.5 Molecular dynamics (MD) simulation study

As both the SW-MLR and GA-MLR models predicted identical SARS-CoV-2 3CL^pro^ inhibitory potency (Table 6), the SARS-CoV-2 3CL^pro^-co-crystallized compound **19** (most active molecule of the dataset) as well as the screened drug Elobixibat (DB12486), both were subjected for further 100ns MD simulation analysis using *DESMOND* module of Schrodinger Maestro v12.1 [30].

**Fig. 11:**
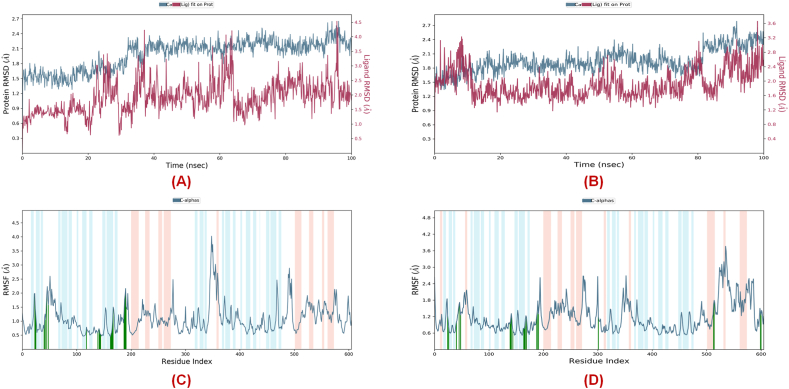
The *RMSD* plots for **(A)** compound **19**, **(B)** Elobixibat and the *RMS* fluctuation of **(C)** compound **19(D)** Elobixibat obtained from the MD simulation study.

From the root mean square deviation (*RMSD*) of the trajectories of each of the P-L complexes (Fig. 11**A** and **B**), it was noticeable that the protein (**PDB ID:** 7L13) demonstrated almost similar fluctuations (<2.7 Å) while complexed with both compound **19** and Elobixibat whereas a comparatively less fluctuation (around 0.9 Å to 3.7 Å) when compared to the co-crystallized compound **19**. On the other hand, regarding the root mean square fluctuation (*RMSF*) of the protein, it was observed that both the catalytic dyads (His41 and Cys145) while complexed with compound **19** showed lower fluctuations (0.72 Å and 0.54 Å, respectively) compared to Elobixibat (1.00 Å and 0.71 Å, respectively). Also, for the other key active site residues such as Gly143, Met165, Glu166, Leu167, Pro168, Gln189, Thr190, and Gln192 residues, a similar fluctuation was noticed for both of these complexes except Gly143 which displayed a comparatively higher fluctuation for the Elobixibat (Gly143 = 1.14 Å) than the co-crystallized compound **19** (Gly143 = 0.67 Å) whereas the S4 pocket-forming residues such as Gln189, Thr190, and Gln192 residues showed less fluctuation in the case of Elobixibat (Gln189 = 1.25 Å, Thr190 = 1.42 Å, and Gln192 = 1.29 Å) than compound **19**, (Gln189 = 1.77 Å, Thr190 = 1.94 Å, and Gln192 = 1.53 Å) (Fig. 11**C** and **D**).

#### 3.5.1 Analysis of the protein-ligand-contacts for the MD simulation study

**Fig. 12:**
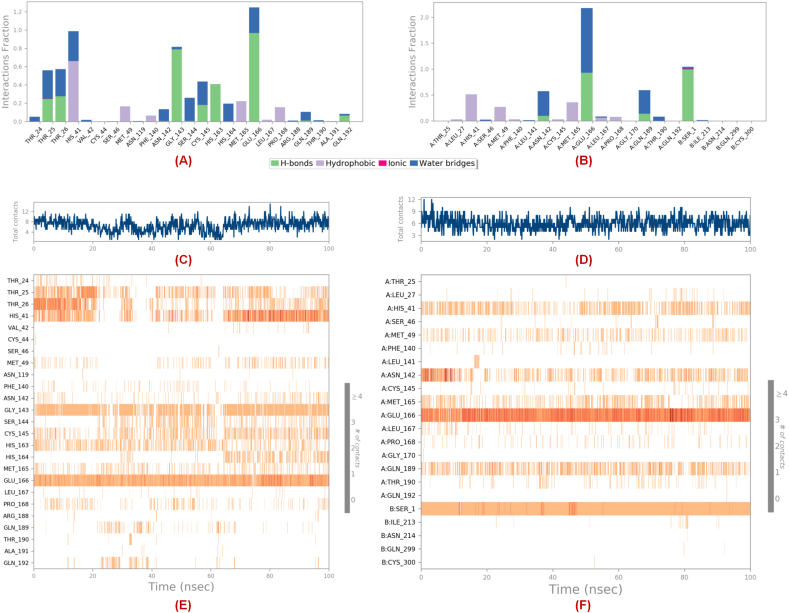
Interaction fraction histogram for **(A)** compound **19** and **(B)** Elobixibat; Total contacts for **(C)** compound **19** and **(D)** Elobixibat; Interaction frequencies for **(E)** compound **19** and **(F)** Elobixibat in complex with SARS-CoV-2 3CL^pro^ (PDB ID: 7L13) obtained from the MD simulation.

Regarding the contacts made by the ligands (compound **19** and Elobixibat) throughout the 100ns MD simulation study explored that the co-crystallized compound **19** showed hydrogen bond interactions with Gly143, Cys145, His163, Glu166, Gln189, and Gln192 (Fig. 12**A**) whilst Elobixibat was able to interact with Asn142, Glu166, and Gln189 residues (Fig. 12**B**). Again, the total contacts made by these simulated compound **19** and Elobixibat at the SARS-CoV-2 3CL^pro^ active site for 100 ns timeframe are shown in Fig. 12**C** and **D**, respectively. Also, both of the molecules formed hydrophobic contacts with Met165, Leu167, Pro168, and Cys165 amino acid residues. At the active site, compound **19** also demonstrated its ability to form better hydrophobic contact with both of the catalytic dyad residues than Elobixibat which can be one of the key reasons behind its potent SARS-CoV-2 3CL^pro^ inhibitory activity. Interestingly, though a higher number of active site residues were found to form water-bridge interactions with compound **19**, the extent of water-bridge interactions between Elobixibat and Glu166, Gln189, and Thr190 residues was comparatively higher. Such a high extent of water-mediated interactions of Elobixibat at the S4 pocket residues explained the comparatively lower *RMSF* observed previously (Fig. 12**E** and **F**).

**Fig. 13:**
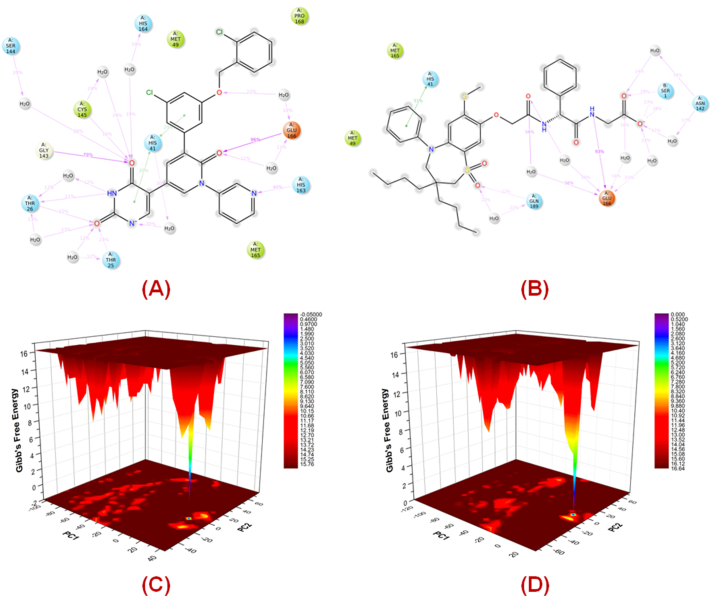
2D interaction summary of **(A)** compound **19**, **(B)** Elobixibat obtained from 100ns MD simulation study; The FEL plots for **(C)** compound **19**, **(D)** Elobixibat.

Therefore, the simulation interaction analysis also suggested the capability of both of these compounds to form good interactions in the dimer form of the protein. Similar observations can be noticed in the interaction frequency analysis where compound **19** showed more frequent contact with the catalytic dyad in comparison to Elobixibat. However, Elobixibat demonstrated its capability not only to form strong and frequent contact with Glu166 near the S1′ pocket as well as Gln189 and Thr190 at the S4 pocket, providing stable binding at the catalytic site. It was a noticeable factor that the oxo function of the pyridinone ring was the only functional group capable of interacting with Glu166 at the S1′ pocket where multiple functional groups of Elobixibat were able to form both hydrogen bonding and water-bridge interactions with Glu166, providing stability and proximity to His41 to form frequent interactions to disrupt its catalytic function (Fig. 13**A** and **B**).

#### 3.5.2 MM/GBSA, trajectory PCA, and free energy landscape (FEL) analysis

The molecular mechanics with generalized Born and surface area solvation (MM/GBSA) is a free binding energy calculation method to evaluate the binding energy of the simulated P-L complexes. To calculate the free binding energy of the simulated P-L complexes, the *PRIME* module and the *thermal_mmgbsa.py* script of Schrodinger Maestro v12.1 software [30] were used with a step-value of 10. The binding free energy values for both the SARS-CoV-2-compound **19** and SARS-CoV-2-Elobixibat complexes are provided in Table 7. It was also interesting to note that though both of the complexes showed a similar range of binding energy (Table 7), Elobixibat was found to be more stable compared to compound **19** in terms of the stability and low binding energy.

**Table 7:** The summary of the MM/GBSA analysis performed for the simulated complexes for a 100ns timeframe.

Complex	Summary
7L13-compound **19** complex	dG Average: -52.4933
	dG Standard Deviation: 32.45
	dG Range: -98.7899 to 3.4788
	dG(NS) Average: -55.3251
	dG(NS) Standard Deviation: 33.99
	dG(NS) Range: -101.2030 to 3.4045
7L13-Elobixibat complex	dG Average: -60.7490
	dG Standard Deviation: 27.17
	dG Range: -88.7709 to 0.4565
	dG(NS) Average: -69.4118
	dG(NS) Standard Deviation: 31.19
	dG(NS) Range: -101.5549 to 0.4619
MMGBSA dG Bind: The binding energy of the receptor and ligand as calculated by	
the Prime Energy, a Molecular Mechanics + Implicit Solvent Energy	
Function (kcals/mol)	
=PrimeEnergy(Optimized Complex)	
- PrimeEnergy(Optimized Free Ligand)	
- PrimeEnergy(Optimized Free Receptor)	
MMGBSA dG Bind(NS): A version of dG Bind that does not include contributions	
from receptor or ligand strain. (kcals/mol)	
=PrimeEnergy(Optimized Complex)	
- PrimeEnergy(Ligand Geometry From Optimized Complex)	
- PrimeEnergy(Receptor Geometry From Optimized Complex)	
Lig Strain Energy: A prediction of the energetic penalty due to strain between	
the ligand in the complex and the ligand in the free state based on the	
difference in Prime Energy (kcals/mol).	
= PrimeEnergy(Ligand Geometry From Optimized Complex)	
- PrimeEnergy(Optimized Free Ligand)	
Rec Strain Energy: A prediction of the energetic penalty due to strain between	
the receptor in the complex and the receptor in the free state based on the difference in Prime Energy (kcals/mol).	
= PrimeEnergy(Receptor Geometry From Optimized Complex)	
- PrimeEnergy(Optimized Free Receptor)	

**Fig. 14:**
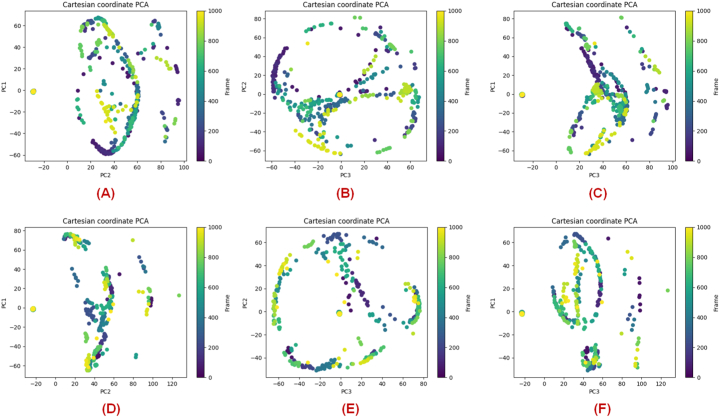
**(A–C)** The trajectory PCA plots for the A-chain of SARS-CoV-2 3CL^pro^ (PDB ID: 7L13) in complex with compound **19; (D**–**F)** The trajectory PCA plots for the A-chain of SARS-CoV-2 3CL^pro^ (PDB ID: 7L13) in complex with Elobixibat.

The trajectory principal component analysis (PCA) and principal component-based Free energy landscape (FEL) analysis for the A chain of the protein simulated in complex with compound **19** and Elobixibat were performed here with the aid of G_Measure v0.9d [49]. For the cumulative PCA analysis, a total of 10 principal components were calculated for the trajectory PCA analysis. The trajectory PCA plots for compound **19** (Fig. 14**A**-14**C**) and Elobixibat (Fig. 14**D**–**F**) were obtained from the analysis in this study of these simulated P-L complexes showed that it can explain 87.49% of the variance in the trajectory with the first 3 components (PC1-PC3) whereas for the Elobixibat the first 3 PCs of the chain A showed an 87.20% explained variance ratio.

The analysis of the free energy landscape (FEL) is a sampling method for the conformations to explore the near-native state conformations along with variability in conformations of the protein via Gibb's free energy [50]. Here, the FEL analysis of the simulated complexes was performed with the help of GROMACS v2020.6 [51,52]. To perform the FEL analysis, the first two principal components (PC1 and PC2) for each of the complexes were calculated using the *g_covar* and *g_anaeig* scripts of GROMACS v2020.6 where the *g_sham* script was used to compute the Gibb's free energy. The FEL plots for both of the complexes (Fig. 13**C** and **D**) showed a stable low-energy conformation (violet to violet-blue cleft) for both of the complexes suggesting the achievement of stable low-energy conformation for the 100ns MD simulation study as it was also noticed in the MM/GBSA analysis that showed stable low-energy conformations were achieved for both of the compounds while complexed with SARS-CoV-2 3CL^pro^ (PDB ID: 7L13).

Therefore, these observations clearly suggested the almost identical binding of Elobixibat in compared to the SARS-CoV-2 3CL^pro^ (PDB ID: 7L13)-co-crystallized compound **19** and nominates Elobixibat as a potential lead to either drug repurposing or to lead modification and drug design to develop a safer and more effective anti-SARS-CoV-2 agent with less toxic effects. Nonetheless, the peptidomimetic-like structure of Elobixibat can also provide insights to further design newer peptidomimetic or non-peptidomimetic drug candidates against SARS-CoV-2 with better drug likeliness.

## 4 Conclusion

**Fig. 15:**
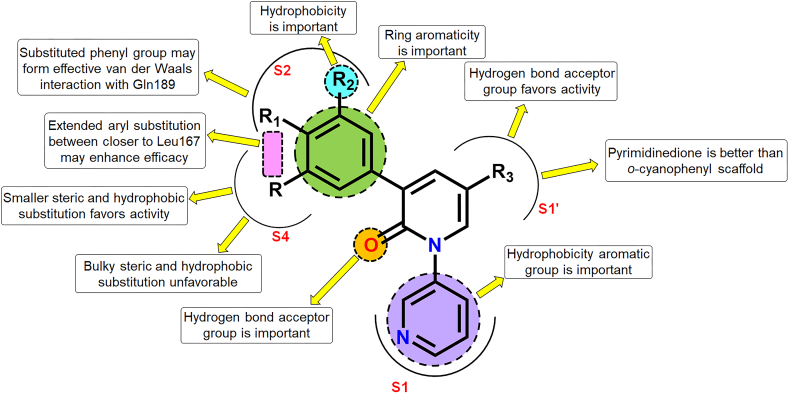
Structure-activity relationship summary of the triaryl pyridinone-based non-covalent SARS-CoV-2 3CL^pro^ inhibitors.

Even after the pandemic situation, the rising number of SARS-CoV-2-related cases and the virus-related mutations are some of the major concerns. Also, the drawbacks of the COVID-19 vaccines and the paucity of orally effective molecules for the treatment of severe SARS-CoV-2 infection require an effective remedy. Furthermore, the significant contribution of the SARS-CoV-2 3CL^pro^ is one of the budding targets for the development of effective small-molecule drug candidates against the infection. Here, the docking-based 2D-QSAR model displayed that van der Waals interactions imparted by crucial modulators of SARS-CoV-2 3CL^pro^ inhibition. Compounds with pyrimidinedione scaffold directed towards the S1′ pocket were better than the respective *o*-cyanophenyl derivatives. Again, phenyl or substituted phenyl ring directed between the S2 and S4 pockets may form effective van der Waals interactions with Gln189 at the S2 pocket. Nevertheless, proper positioning of the extended aryl substitution between these S2 and S4 pockets may also be responsible for the higher activity as a closer distance from Leu167 at the S4 pocket may enhance the efficacy. Moreover, a closer distance from Gln192 at the S4 pocket may also favour inhibitory activity. Interestingly, the CoMFA study pointed out the importance of both favourable and unfavourable steric contours near the S4 subsite. It showed that smaller groups with steric effects may be favourable at this position but bulky substitutions with greater steric effects may be unfavourable at this position. Nevertheless, the CoMSIA study also revealed the importance of hydrophobicity at the S4 position. The importance of hydrophobicity in this position was also supported by the common feature pharmacophore mapping analysis. Combining all these observations, it may be presumed that the S4 pocket is such an important area of the SARS-CoV-2 3CL^pro^ enzyme where the importance of both the steric and hydrophobic features was noticed. However, substitutions at the aryl group at this position should be done in such a way that it should be smaller in size. If it is bulky then it may produce a negative impact on SARS-CoV-2 3CL^pro^ inhibition. Therefore, smaller steric and hydrophobic substitution at the S4 site is necessary to impart the higher inhibitory effects. On the other hand, the common feature pharmacophore mapping revealed that hydrogen bond acceptor groups are favourable at S1′ and in between S1 and S2 groups. Moreover, a ring aromatic feature along with a hydrophobic feature is essential at the S2 subsite for exerting higher SARS-CoV-2 3CL^pro^ inhibition. The crucial structural attributes responsible for exerting potential SARS-CoV-2 3CL^pro^ inhibitory activity are depicted in Fig. 15. Taking into consideration all these crucial attributes, potential and highly effective inhibitors of SARS-CoV-2 3CL^pro^ can be designed in the future.

Nevertheless, depending on the structure-based virtual screening, two drug candidates were identified as potential leads against SARS-CoV-2 3CL^pro^. Among these compounds, the repurposed drug Elobixibat which is used for the management of constipation and can be utilized as a potential non-covalent effective agent for the treatment of COVID-19 with fewer adverse effects as suggested by the current molecular modeling studies.

## CRediT authorship contribution statement

**Suvankar Banerjee:** Writing – original draft, Validation, Methodology, Investigation, Formal analysis, Data curation. **Sandip Kumar Baidya:** Writing – review & editing, Visualization, Validation, Methodology. **Balaram Ghosh:** Writing – review & editing, Visualization, Software, Methodology. **Tarun Jha:** Writing – review & editing, Visualization, Validation. **Nilanjan Adhikari:** Writing – review & editing, Validation, Supervision, Project administration, Investigation, Conceptualization.

## Data availability statement

No additional data was used for the research described in the article.

## Declaration of competing interest

The authors declare that they have no known competing financial interests or personal relationships that could have appeared to influence the work reported in this paper.

## References

[1] Nicola M., Alsafi Z., Sohrabi C., Kerwan A., Al-Jabir A., Iosifidis C., Agha M., Agha R. (2020). The socio-economic implications of the coronavirus pandemic (COVID-19): a review. Int. J. Surg..

[2] Karunathilake K. (2020). Positive and negative impacts of COVID-19, an analysis with special reference to challenges on the supply chain in South Asian countries. J. Econ. Soc. Dev..

[3] Mulugeta T., Tadesse E., Shegute T., Desta T.T. (2021). COVID-19: socio-economic impacts and challenges in the working group. Heliyon.

[4] Murphy K. (2020). SARS CoV-2 Detection from upper and lower respiratory tract specimens: diagnostic and infection control implications. Chest.

[5] https://www.worldometers.info/coronavirus/, as accessed in December 2022.

[6] Mody V., Ho J., Wills S., Mawri A., Lawson L., Ebert M., Fortin G.M., Rayalam S., Taval S. (2021). Identification of 3-chymotrypsin like protease (3CL^Pro^) inhibitors as potential anti-SARS-CoV-2 agents. Commun. Biol..

[7] Wu F., Zhao S., Yu B., Chen Y.M., Wang W., Song Z.G., Hu Y., Tao Z.W., Tian J.H., Pei Y.Y., Yuan M.L., Zhang Y.L., Dai F.H., Liu Y., Wang Q.M., Zheng J.J., Xu L., Holmes E.C., Zhang Y.Z. (2020). A new coronavirus associated with human respiratory disease in China. Nature.

[8] Tahir Ul Qamar M., Alqahtani S.M., Alamri M.A., Chen L.L. (2020). Structural basis of SARS-CoV-2 3CLpro and anti-COVID-19 drug discovery from medicinal plants. J. Pharm. Anal..

[9] Adhikari N., Banerjee S., Baidya S.K., Ghosh B., Jha T. (2021). Robust classification-based molecular modelling of diverse chemical entities as potential SARS-CoV-2 3CLpro inhibitors: theoretical justification in light of experimental evidences, SAR QSAR Env. Res..

[10] Artika I.M., Dewantari A.K., Wiyatno A. (2020). Molecular biology of coronaviruses: current knowledge. Heliyon.

[11] Banerjee S., Baidya S.K., Adhikari N., Ghosh B., Jha T. (2023). Glycyrrhizin as a promising kryptonite against SARS-CoV-2: clinical, experimental, and theoretical evidences. J. Mol. Struct..

[12] Ferreira J.C., Fadl S., Villanueva A.J., Rabeh W.M. (2021). Catalytic dyad residues His41 and Cys145 impact the catalytic activity and overall conformational fold of the main SARS-CoV-2 protease 3-chymotrypsin-like protease. Front. Chem..

[13] Fernandes H.S., Sousa S.F., Cerqueira N.M.F.S.A. (2022). New insights into the catalytic mechanism of the SARS-CoV-2 main protease: an ONIOM QM/MM approach. Mol. Divers..

[14] Ma C., Sacco M.D., Hurst B., Townsend J.A., Hu Y., Szeto T., Zhang X., Tarbet B., Marty M.T., Chen Y., Wang J. (2020). Boceprevir, GC-376, and calpain inhibitors II, XII inhibit SARS-CoV-2 viral replication by targeting the viral main protease. Cell Res..

[15] Amin S.A., Banerjee S., Singh S., Qureshi I.A., Gayen S., Jha T. (2021). First structure–activity relationship analysis of SARS-CoV-2 virus main protease (Mpro) inhibitors: an endeavor on COVID-19 drug discovery. Mol. Divers..

[16] Wang M., Cao R., Zhang L., Yang X., Liu J., Xu M., Shi Z., Hu Z., Zhong W., Xiao G. (2020). Remdesivir and chloroquine effectively inhibit the recently emerged novel coronavirus (2019-nCoV) in vitro. Cell Res..

[17] De P., Chakraborty I., Karna B., Mazumder N. (2021). Brief review on repurposed drugs and vaccines for possible treatment of COVID-19. Eur. J. Pharmacol..

[18] Harrison C. (2020). Coronavirus puts drug repurposing on the fast track. Nat. Biotechnol..

[19] Chakraborty C., Sharma A.R., Bhattacharya M., Agoramoorthy G., Lee S.S. (2021). The drug repurposing for COVID-19 clinical trials provide very effective therapeutic combinations: lessons learned from major clinical studies. Front. Pharmacol..

[20] Srivastava K., Singh M.K. (2021). Drug repurposing in COVID-19: a review with past, present and future. Metabol. Open.

[21] Yates P.A., Newman S.A., Oshry L.J., Glassman R.H., Leone A.M., Reichel E. (2020). Doxycycline treatment of high-risk COVID-19-positive patients with comorbid pulmonary disease. Ther. Adv. Respir. Dis..

[22] Wishart D.S., Knox C., Guo A.C., Shrivastava S., Hassanali M., Stothard P., Chang Z., Woolsey J. (2006). DrugBank: a comprehensive resource for in silico drug discovery and exploration. Nucleic Acids Res..

[23] Zhang C.H., Spasov K.A., Reilly R.A., Hollander K., Stone E.A., Ippolito J.A., Liosi M.E., Deshmukh M.G., Tirado-Rives J., Zhang S., Liang Z., Miller S.J., Isaacs F., Lindenbach B.D., Anderson K.S., Jorgensen W.L. (2021). Optimization of triarylpyridinone inhibitors of the main protease of SARS-CoV-2 to low-nanomolar antiviral potency. ACS Med. Chem. Lett..

[24] Deshmukh M.G., Ippolito J.A., Zhang C.H., Stone E.A., Reilly R.A., Miller S.J., Jorgensen W.L., Anderson K.S. (2021). Structure-guided design of a perampanel-derived pharmacophore targeting the SARS-CoV-2 main protease. Structure.

[25] Zhang C.H., Stone E.A., Deshmukh M., Ippolito J.A., Ghahremanpour M.M., Tirado-Rives J., Spasov K.A., Zhang S., Takeo Y., Kudalkar S.N., Liang Z., Isaacs F., Lindenbach B., Miller S.J., Anderson K.S., Jorgensen W.L. (2021). Potent noncovalent inhibitors of the main protease of SARS-COV-2 from molecular sculpting of the drug perampanel guided by free energy perturbation calculations. ACS Cent. Sci..

[26] (2010). ChemDraw Ultra 5.0.

[27] (2015). Discovery Studio 3.0 (DS 3.0).

[28] Banerjee S., Baidya S.K., Ghosh B., Adhikari N., Jha T. (2022). The first report on predictive comparative ligand-based multi-QSAR modeling analysis of 4-pyrimidinone and 2-pyridinone based APJ inhibitors. New J. Chem..

[29] RCSB Protein Data Bank, https://www.rcsb.org/, as accessed in August 2022.

[30] (2019). Schrodinger Suite.

[31] Jorgensen W.L., Maxwell D.S., TiradoRives J. (1996). Development and testing of the OPLS ALL-ATOM force field on conformational energetics and properties of organic liquids. J. Am. Chem. Soc..

[32] Tamang J.S.D., Banerjee S., Baidya S.K., Ghosh B., Adhikari N., Jha T. (2024). Employing comparative QSAR techniques for the recognition of dibenzofuran and dibenzothiophene derivatives toward MMP-12 inhibition. J. Biomol. Struct. Dyn..

[33] Adhikari N., Amin S.A., Saha A., Jha T. (2017). Understanding chemico-biological interactions of glutamate MMP-2 inhibitors through rigorous alignment-dependent 3D-QSAR analyses. ChemistrySelect.

[34] (2022). The simple, user-friendly and reliable online standalone tools freely. http://teqip.jdvu.ac.in/QSAR_Tools/.

[35] Adhikari N., Banerjee S., Baidya S.K., Ghosh B., Jha T. (2022). Ligand-based quantitative structural assessments of SARS-CoV-2 3CL^pro^ inhibitors: an analysis in light of structure-based multi-molecular modeling evidences. J. Mol. Struct..

[36] Golbraikh A., Tropsha A. (2002). Beware of q2. J. Mol. Graph. Model..

[37] Roy K., Chakraborty P., Mitra I., Ojha P.K., Kar S., Das R.N. (2013). Some case studies on application of "r(m)2" metrics for judging quality of quantitative structure-activity relationship predictions: emphasis on scaling of response data. J. Comput. Chem..

[38] Yadav V., Banerjee S., Baidya S.K., Adhikari N., Jha T. (2022). Applying comparative molecular modelling techniques on diverse hydroxamate-based HDAC2 inhibitors: an attempt to identify promising structural features for potent HDAC2 inhibition. SAR QSAR Environ. Res..

[39] Adhikari N., Halder A.K., Mondal C., Jha T. (2013). Exploring structural requirements of aurone derivatives as antimalarials by validated DFT-based QSAR, HQSAR, and COMFA-COMSIA approach. Med. Chem. Res..

[40] Cramer R.D., Patterson D.E., Bunce J.D. (1988). Comparative molecular field analysis (CoMFA). 1. Effect of shape on binding of steroids to carrier proteins. J. Am. Chem. Soc..

[41] Verma J., Khedkar V.M., Coutinho E.C. (2010). 3D-QSAR in drug design--a review. Curr. Top. Med. Chem..

[42] Klebe G., Abraham U. (1999). Comparative molecular similarity index analysis (CoMSIA) to study hydrogen-bonding properties and to score combinatorial libraries. J. Comput. Aided Mol. Des..

[43] (2012). SYBYL-X 2.0 Software.

[44] Ataei S., Yilmaz S., Ertan-Bolelli T., Yildiz I. (2015). Generated 3D-common feature hypotheses using the HipHop method for developing new topoisomerase I inhibitors. Arch. Pharm..

[45] Adane L., Bharatam P.V., Sharma V.A. (2010). Common feature-based 3D-pharmacophore model generation and virtual screening: identification of potential PfDHFR inhibitors. J. Enzym. Inhib. Med. Chem..

[46] Uba A.I., Yelekçi K. (2018). Pharmacophore-based virtual screening for identification of potential selective inhibitors of human histone deacetylase 6. Comput. Biol. Chem..

[47] http://dude.docking.org/, as accessed in October 2022.

[48] Mysinger M.M., Carchia M., Irwin J.J., Shoichet B.K. (2012). Directory of useful decoys, enhanced (DUD-E): better ligands and decoys for better benchmarking. J. Med. Chem..

[49] Kagami L.P., das Neves G.M., Timmers L.F.S.M., Caceres R.A., Eifler-Lima V.L. (2020). Geo-Measures: a PyMOL plugin for protein structure ensembles analysis. Comput. Biol. Chem..

[50] Banerjee S., Jana S., Jha T., Ghosh B., Adhikari N. (2024). An assessment of crucial structural contributors of HDAC6 inhibitors through fragment-based non-linear pattern recognition and molecular dynamics simulation approaches. Comput. Biol. Chem..

[51] Groningen machine for chemical simulations; Software available at: https://www.gromacs.org/.As accessed in October 2024.

[52] Páll S., Zhmurov A., Bauer P., Abraham M., Lundborg M., Gray A., Hess B., Lindahl E. (2020). Heterogeneous parallelization and acceleration of molecular dynamics simulations in GROMACS. J. Chem. Phys..

