# Supplementary material for: Exploring the key structural attributes and chemico-biological interactions of pyridinone-based SARS-CoV-2 3CLpro inhibitors through validated structure-based drug design strategies
Source: Heliyon. 2024 Nov 15;10(23):e40404. doi: 10.1016/j.heliyon.2024.e40404 (PMC11626027; doi:10.1016/j.heliyon.2024.e40404)
Supplement: Multimedia component 1 [file mmc1.docx]

**Supplementary material**

**Exploring the key structural attributes and chemico-biological interactions of pyridinone-based SARS-CoV-2 3CL^pro^ inhibitors through validated structure-based drug design strategies**

Suvankar Banerjee ^a^, Sandip Kumar Baidya ^a^, Balaram Ghosh ^b^, Tarun Jha ^a^, Nilanjan Adhikari ^a, *^

*^a^Natural Science Laboratory, Division of Medicinal and Pharmaceutical Chemistry, Department of Pharmaceutical Technology, Jadavpur University, Kolkata 700032, India*

*^b^Epigenetic Research Laboratory, Department of Pharmacy, Birla Institute of Technology and Science-Pilani, Hyderabad Campus, Shamirpet, Hyderabad, 500078, India*

Corresponding author:

Nilanjan Adhikari ([nilanjan_juphar@rediffmail.com](mailto:nilanjan_juphar@rediffmail.com))

**Supplementary Table 1.** Structure and SARS-CoV-2 3CL^Pro^ inhibitory activity the dataset compounds


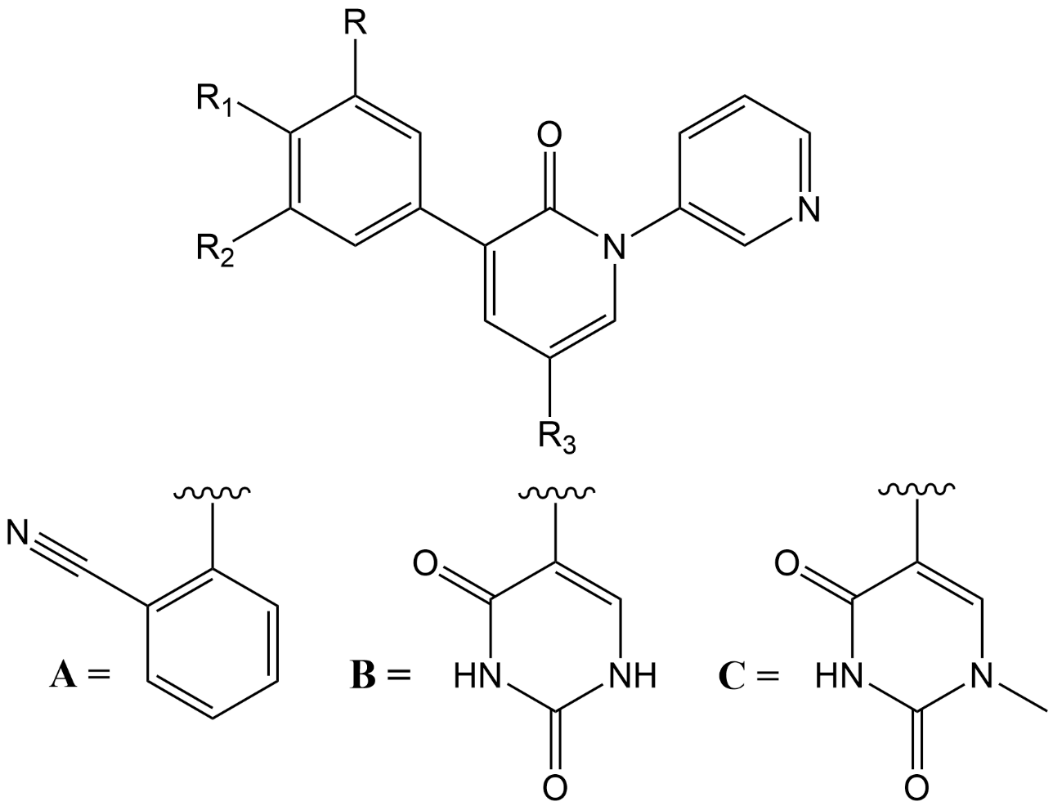


| **Sl No** | **R** | **R_1_** | **R_2_** | **R_3_** | **IC_50_ (µM)** | **pIC_50_** |
| --- | --- | --- | --- | --- | --- | --- |
| 1 | H | H | Cl | A | 9.99 | 5.000 |
| 2 | H | H | Cl | B | 6.38 | 5.195 |
| 3* | Cl | H | Cl | A | 4.02 | 5.396 |
| 4 | O-C_3_H_7_ | H | Cl | A | 0.14 | 6.854 |
| 5 | O-C_2_H_4_-O-CH_3_ | H | Cl | A | 0.47 | 6.328 |
| 6 | O-Bnz | H | Cl | A | 0.28 | 6.553 |
| 7 | Cl | H | O-CH_2_-Bnz | A | 0.51 | 6.292 |
| 8 | O-CH_3_ | H | Cl | B | 1.2 | 5.921 |
| 9 | O-C_3_H_7_ | H | Cl | B | 0.12 | 6.921 |
| 10 | O-C_4_H_9_ | H | Cl | B | 0.25 | 6.602 |
| 11* | Cl | H | O-*i*-Pentyl | B | 0.19 | 6.721 |
| 12 | O-Bnz | H | Cl | B | 0.128 | 6.893 |
| 13 | O-CH_2_-Bnz | H | Cl | B | 0.11 | 6.959 |
| 14 | O-*o*-CH_3_-Bnz | H | Cl | B | 0.1 | 7.000 |
| 15* | O-*m*-CH_3_-Bnz | H | Cl | B | 0.11 | 6.959 |
| 16 | O-*o*-F-Bnz | H | Cl | B | 0.024 | 7.620 |
| 17 | O-*m*-F-Bnz | H | Cl | B | 0.037 | 7.432 |
| 18 | O- 2,6-difluorobenzyl | H | Cl | B | 0.036 | 7.444 |
| 19 | O-*o*-Cl-Bnz | H | Cl | B | 0.018 | 7.745 |
| 20 | O-2Cl-4F-Bnz | H | Cl | B | 0.036 | 7.444 |
| 21 | O-*o*-Cl-Bnz | F | Cl | B | 0.02 | 7.699 |
| 22* | O-CH_2_-cyclopropyl | H | Cl | B | 0.037 | 7.432 |
| 23* | O-C_2_H_4_-CF_3_ | H | Cl | B | 0.025 | 7.602 |
| 24 | O-CH_2_-cyclopropyl | H | Cl | A | 0.17 | 6.770 |
| 25 | O-C_2_H_4_-CF_3_ | H | Cl | A | 0.12 | 6.921 |
| 26* | O-CH_2_-cyclopentyl | H | Cl | A | 0.328 | 6.484 |
| 27 | O-CH_2_-5-methyloxazole | H | Cl | A | 0.083 | 7.081 |
| 28* | O-CH_2_-5-methyloxazole | H | Cl | B | 0.085 | 7.071 |
| 29 | O-CH_2_-4-methyloxazole | H | Cl | B | 0.131 | 6.883 |
| 30 | O-CH_2_-2-Cl-thiophen | H | Cl | B | 0.027 | 7.569 |
| 31 | O-CH_2_-5-methylthiazole | H | Cl | B | 0.042 | 7.377 |
| 32 | O-CH_2_-4-CF_3_-oxazole | H | Cl | B | 0.105 | 6.979 |
| 33 | O-CH_2_-4-CF_3_-thiazole | H | Cl | B | 0.038 | 7.420 |
| 34 | O-CH_2_-cyclopropyl | H | Cl | C | 0.061 | 7.215 |
| 35* | O-C_2_H_4_-CF_3_ | H | Cl | C | 0.059 | 7.229 |
| 36 | O-*o*-Cl-Bnz | F | Cl | C | 0.044 | 7.357 |
| 37* | O-CH_2_-4CF_3_-oxazole | H | Cl | C | 0.13 | 6.886 |
| 38 | O-CH_2_-4CF_3_-thiazole | H | Cl | C | 0.061 | 7.215 |

* marked compounds are considered as the test set compounds

**Supplementary Table 2.** Values of the final features used for the SW-MLR and GA-MLR models

| ***Cpd No*** | ***pIC_50_ (M)*** | ***SW-MLR (Equation 1)*** | | | ***GA-MLR (Equation 2)*** | | |
| --- | --- | --- | --- | --- | --- | --- | --- |
|  |  | ***r_glide_res:A41_vdw*** | ***r_glide_res:A167_dist*** | ***r_glide_res:A189_vdw*** | ***r_glide_res:A41_vdw*** | ***r_i_glide_evdw*** | ***r_glide_res:A192_dist*** |
| ***Training set*** | | | | | | | |
| 1 | 5.000 | -4.735 | 5.55098 | -1.82025 | -4.735 | -50.774 | 4.74557 |
| 2 | 5.195 | -4.83141 | 5.63787 | -1.96153 | -4.83141 | -50.7463 | 4.70268 |
| 4 | 6.854 | -4.83054 | 2.20372 | -3.48986 | -4.83054 | -57.7213 | 2.22578 |
| 5 | 6.328 | -4.89872 | 3.38025 | -3.73312 | -4.89872 | -55.9128 | 2.16837 |
| 6 | 6.553 | -5.01332 | 2.38132 | -4.54574 | -5.01332 | -61.7859 | 2.05791 |
| 7 | 6.292 | -3.87801 | 3.16667 | -5.65543 | -3.87801 | -59.0882 | 3.02157 |
| 8 | 5.921 | -4.78952 | 3.82735 | -3.18349 | -4.78952 | -53.7509 | 3.36125 |
| 9 | 6.921 | -4.95293 | 3.28873 | -3.54227 | -4.95293 | -57.8828 | 3.53097 |
| 10 | 6.602 | -4.99973 | 1.97885 | -3.10299 | -4.99973 | -58.3295 | 2.22431 |
| 12 | 6.893 | -4.72466 | 3.88307 | -5.25408 | -4.72466 | -62.9961 | 2.25001 |
| 13 | 6.959 | -4.62179 | 2.34915 | -3.75699 | -4.62179 | -63.9269 | 2.11043 |
| 14 | 7.000 | -4.9167 | 2.81173 | -4.72453 | -4.9167 | -64.5916 | 2.11926 |
| 16 | 7.620 | -5.0657 | 3.11222 | -4.58347 | -5.0657 | -63.6679 | 2.04392 |
| 17 | 7.432 | -4.93078 | 2.08497 | -4.36782 | -4.93078 | -66.224 | 2.2469 |
| 18 | 7.444 | -4.72576 | 2.35296 | -4.67981 | -4.72576 | -63.5511 | 2.06067 |
| 19 | 7.745 | -4.92127 | 2.78638 | -4.54811 | -4.92127 | -64.9639 | 2.1247 |
| 20 | 7.444 | -5.16915 | 3.35253 | -5.13129 | -5.16915 | -63.516 | 2.09257 |
| 21 | 7.699 | -4.93009 | 2.83072 | -4.48776 | -4.93009 | -63.9632 | 2.25503 |
| 24 | 6.770 | -5.18644 | 2.90381 | -3.80683 | -5.18644 | -56.3403 | 2.71996 |
| 25 | 6.921 | -4.85721 | 2.76248 | -4.01019 | -4.85721 | -59.3697 | 2.26289 |
| 27 | 7.081 | -4.96851 | 3.86522 | -5.1794 | -4.96851 | -61.249 | 2.5965 |
| 29 | 6.883 | -4.94985 | 2.06737 | -5.10855 | -4.94985 | -65.3427 | 2.64926 |
| 30 | 7.569 | -4.97549 | 3.77485 | -5.7975 | -4.97549 | -62.7884 | 2.19766 |
| 31 | 7.377 | -4.92186 | 3.65243 | -5.79422 | -4.92186 | -64.8098 | 3.12085 |
| 32 | 6.979 | -4.94844 | 3.72391 | -5.70691 | -4.94844 | -65.254 | 2.43115 |
| 33 | 7.420 | -4.65895 | 2.56134 | -5.35658 | -4.65895 | -65.4279 | 2.21213 |
| 34 | 7.215 | -5.26346 | 2.64427 | -3.44307 | -5.26346 | -58.9503 | 3.53592 |
| 36 | 7.357 | -4.96724 | 3.15535 | -4.48878 | -4.96724 | -64.7023 | 2.43847 |
| 38 | 7.215 | -5.03414 | 3.82493 | -6.07278 | -5.03414 | -67.7933 | 2.75265 |
| ***Test Set*** | | | | | | | |
| 3 | 5.396 | -4.89956 | 5.6077 | -2.61845 | -4.89956 | -49.7069 | 4.52072 |
| 11 | 6.721 | -4.97885 | 2.30353 | -4.25044 | -4.97885 | -58.3466 | 3.6571 |
| 15 | 6.959 | -4.9382 | 2.3259 | -4.69098 | -4.9382 | -65.3352 | 2.06203 |
| 22 | 7.432 | -4.9505 | 2.17817 | -3.79402 | -4.9505 | -59.3658 | 1.92763 |
| 23 | 7.602 | -4.89308 | 2.57965 | -3.9077 | -4.89308 | -61.6562 | 2.29636 |
| 26 | 6.484 | -4.34686 | 2.24841 | -4.52988 | -4.34686 | -61.7 | 1.67112 |
| 28 | 7.071 | -4.95074 | 3.51363 | -5.10888 | -4.95074 | -64.6176 | 2.2473 |
| 35 | 7.229 | -4.90031 | 2.5722 | -3.95924 | -4.90031 | -60.467 | 2.29701 |
| 37 | 6.886 | -4.66598 | 2.70336 | -5.33383 | -4.66598 | -65.1492 | 2.24817 |

**Supplementary Table 3.** Actual and predicted activities for the MLR and CoMFA, CoMSIA-models

| ***Cpd No*** | ***Observed***  ***pIC_50_*** | ***Predicted pIC_50_*** | | | |
| --- | --- | --- | --- | --- | --- |
|  |  | ***SW-MLR*** | ***GA-MLR*** | ***CoMFA*** | ***CoMSIA*** |
| ***Training Set*** | | | | | |
| 1 | 5.000 | 5.097 | 5.338 | 4.820 | 4.842 |
| 2 | 5.195 | 5.210 | 5.400 | 5.205 | 5.275 |
| 4 | 6.854 | 6.836 | 6.670 | 6.777 | 6.495 |
| 5 | 6.328 | 6.624 | 6.556 | 6.491 | 6.454 |
| 6 | 6.553 | 7.338 | 7.189 | 6.506 | 6.681 |
| 7 | 6.292 | 6.475 | 6.058 | 6.228 | 6.345 |
| 8 | 5.921 | 6.183 | 5.993 | 6.020 | 6.143 |
| 9 | 6.921 | 6.631 | 6.423 | 6.871 | 6.990 |
| 10 | 6.602 | 6.916 | 6.821 | 6.885 | 6.941 |
| 12 | 6.893 | 6.874 | 7.090 | 7.093 | 6.941 |
| 13 | 6.959 | 6.700 | 7.153 | 7.012 | 6.961 |
| 14 | 7.000 | 7.183 | 7.378 | 7.321 | 7.293 |
| 16 | 7.620 | 7.173 | 7.396 | 7.413 | 7.403 |
| 17 | 7.432 | 7.289 | 7.504 | 7.204 | 7.243 |
| 18 | 7.444 | 7.135 | 7.189 | 7.319 | 7.444 |
| 19 | 7.745 | 7.130 | 7.413 | 7.517 | 7.546 |
| 20 | 7.444 | 7.396 | 7.428 | 7.422 | 7.433 |
| 21 | 7.699 | 7.102 | 7.293 | 7.535 | 7.565 |
| 24 | 6.770 | 7.060 | 6.618 | 6.814 | 6.705 |
| 25 | 6.921 | 6.880 | 6.827 | 6.946 | 7.061 |
| 27 | 7.081 | 7.073 | 6.978 | 7.076 | 7.213 |
| 29 | 6.883 | 7.585 | 7.331 | 6.999 | 6.755 |
| 30 | 7.569 | 7.336 | 7.225 | 7.410 | 7.291 |
| 31 | 7.377 | 7.324 | 7.147 | 7.364 | 7.241 |
| 32 | 6.979 | 7.294 | 7.377 | 7.118 | 7.081 |
| 33 | 7.420 | 7.260 | 7.286 | 7.440 | 7.596 |
| 34 | 7.215 | 7.076 | 6.695 | 7.197 | 7.009 |
| 36 | 7.357 | 7.036 | 7.335 | 7.500 | 7.589 |
| 38 | 7.215 | 7.475 | 7.578 | 7.185 | 7.155 |
| ***Test Set*** | | | | | |
| 3 | 5.396 | 5.524 | 5.389 | 5.325 | 5.034 |
| 11 | 6.721 | 7.221 | 6.448 | 6.965 | 7.179 |
| 15 | 6.959 | 7.340 | 7.473 | 6.949 | 7.127 |
| 22 | 7.432 | 7.065 | 6.964 | 7.106 | 6.971 |
| 23 | 7.602 | 6.931 | 7.049 | 7.340 | 7.517 |
| 26 | 6.484 | 6.768 | 6.904 | 6.798 | 6.822 |
| 28 | 7.071 | 7.139 | 7.367 | 7.113 | 7.000 |
| 35 | 7.229 | 6.959 | 6.944 | 7.568 | 7.422 |
| 37 | 6.886 | 7.214 | 7.255 | 7.391 | 7.655 |
